# GPX3 Overexpression Ameliorates Cardiac Injury Post Myocardial Infarction Through Activating LSD1/Hif1α Axis

**DOI:** 10.1111/jcmm.70398

**Published:** 2025-02-03

**Authors:** Qi‐Qi Jiang, Chong Du, Ling‐Ling Qian, Tian‐Kai Shan, Yu‐Lin Bao, Ling‐Feng Gu, Si‐Bo Wang, Tong‐Tong Yang, Liu‐Hua Zhou, Ze‐Mu Wang, Ye He, Qi‐Ming Wang, Hao Wang, Ru‐Xing Wang, Lian‐Sheng Wang

**Affiliations:** ^1^ Department of Cardiology The First Affiliated Hospital of Nanjing Medical University Nanjing China; ^2^ Department of Cardiology, The Affiliated Wuxi People's Hospital of Nanjing Medical University, Wuxi People's Hospital, Wuxi Medical Center Nanjing Medical University Wuxi China

**Keywords:** cardiomyocyte apoptosis, GPX3, Hif1α, LSD1, myocardial infarction, oxidative stress

## Abstract

Myocardial infarction (MI) often results in significant loss of cardiomyocytes (CMs), contributing to adverse ventricular remodelling and heart failure. Therefore, promoting CM survival during the acute stage of MI is crucial. This study aimed to investigate the potential role of GPX3 in cardiac repair following MI. First, plasma GPX3 levels were measured in patients with acute MI (AMI), and myocardial GPX3 expression was assessed in a mouse MI model. Furthermore, the effects of GPX3 on MI were investigated through CM‐specific overexpression or knockdown in vitro and in vivo models. RNA sequencing and subsequent experiments were performed to uncover the molecular mechanisms underlying GPX3‐related effects. Multi‐omics database analysis and experimental verification revealed a significant upregulation of GPX3 expression in ischemic myocardium following MI and in CMs exposed to oxygen–glucose deprivation (OGD). Immunofluorescence results further confirmed elevated cytoplasmic GPX3 expression in CMs under hypoxic conditions. In vitro, GPX3 overexpression mitigated reactive oxygen species (ROS) production and enhanced CM survival during hypoxia, while GPX3 knockdown inhibited these processes. In vivo, CM‐specific GPX3 overexpression in the infarct border zone significantly attenuated CM apoptosis and alleviated myocardial injury, promoting cardiac repair and long‐term functional recovery. Mechanistically, GPX3 overexpression upregulated LSD1 and Hif1α protein expression, and rescue experiments confirmed the involvement of the LSD1/Hif1α pathway in mediating the protective effects of GPX3. Overall, our findings suggest that GPX3 exerts a protective role in ischemic myocardium post‐MI, at least partially through the LSD1/Hif1α axis, highlighting its potential as a therapeutic target for MI treatment.

**Abbreviations**
Ad5: adenovirus vector 5
AMI: acute myocardial infarction
cTNT: cardiac troponin T
EF: ejection fraction
FDR: false discovery rate
FS: fractional shortening
GPX3: glutathione peroxidase 3
Hif1α: hypoxia‐inducible factor 1‐alpha
LAD: left anterior descending
LSD1: lysine‐specific demethylase 1
MI: myocardial infarction
MMP2: matrix metalloproteinase 2
MOI: multiplicity of infection
NRCM: neonatal rat cardiomyocyte
OGD: glucose oxygen deprivation
qRT‐PCR: quantitative real‐time polymerase chain reaction
TUNEL: Terminal deoxynucleotidyl transferase‐mediated dUTP in situ nick end labelling
α‐SMA: α‐smooth muscle actin

## 1 Introduction

Acute myocardial infarction (AMI) is a highly fatal ischaemic heart disease that often leads to detrimental outcomes such as heart failure [1]. Following AMI, various changes including increased oxidative stress, microcirculatory disturbances and cellular apoptosis lead to the loss of cardiomyocytes (CMs). This cascade of events triggers a series of pathological consequences and ultimately, cardiac dysfunction. Despite significant advancements in interventional, surgical and pharmacological treatments that have markedly improved patient outcomes, ongoing research is imperative to further elucidate the mechanisms of cardiac injury and to identify novel therapeutic targets for combating AMI‐induced damage [2].

Glutathione peroxidase 3 (GPX3), a glycosylated selenocysteine protein and a member of the GPX family, exhibits potent antioxidative properties. GPX3 catalyses the reduction of glutathione (GSH) to oxidised glutathione (GSSG), effectively ameliorating hydrogen peroxide‐induced cellular damage [3, 4, 5]. Beyond its antioxidative role, emerging evidence suggests that GPX3 is implicated in a variety of biological processes, including cell proliferation, apoptosis, immunity, fibrosis, thrombosis and tumour growth [5, 6, 7, 8, 9, 10, 11]. Notably, clinical observations have linked fluctuations in GPX3 expression with disease progression [7, 8, 12, 13, 14]. Despite these findings, the specific role of GPX3 in cardiac diseases, particularly in myocardial infarction, remains underexplored and warrants further investigation.

Hypoxia‐inducible factor 1 (Hif1), a pivotal regulator of the cellular adaptive response to low‐oxygen environments, is a heterodimer composed of a constitutively expressed β‐subunit (Hif1β) and one of three oxygen‐responsive α‐subunits (Hif1α, Hif2α or Hif3α) [15, 16]. Among these, Hif1α is the most extensively studied subunit within the HIF‐1 complex. Under normoxic conditions, the detection of Hif1α is challenging due to its rapid proteasomal degradation mediated by the ubiquitin‐proteasome pathway. This degradation process is inhibited under hypoxic conditions, allowing Hif1α to accumulate, stabilise and subsequently dimerise with Hif1β. This dimerisation initiates the formation of the active Hif1 complex, which translocates to the nucleus to activate the transcription of a broad array of genes involved in maintaining oxygen homeostasis [17, 18]. The stability of Hif1α, is regulated by various post‐translational modifications (PTMs), which is essential for the survival and metabolic adaptation of cardiomyocytes (CMs) in hypoxic environments [19, 20, 21, 22]. Previous research has suggested that GPX3 may promote the expression of Hif1α; however, the regulatory role of GPX3 on Hif1α in hypoxic CMs and myocardium needs to be further explored.

Lysine‐specific demethylase 1 (LSD1), also known as AOF2/KDM1A, is the first identified lysine‐specific demethylase. Based on its demethylase activity and its nonclassical function, LSD1 has been implicated in a spectrum of physiological and pathological processes, particularly influencing cell survival, growth and differentiation [23, 24]. Recent studies further underscore LSD1's expansive epigenetic regulatory functions, extending its activity to nonhistone substrates, such as Hif1α [24, 25, 26]. Under hypoxic conditions, LSD1 employs a multitude of mechanisms to augment the stability of the Hif1α protein, thereby facilitating cellular adaptation to low‐oxygen environments [26, 27, 28, 29]. This interaction between LSD1 and Hif1α suggests a sophisticated synergy that is critical for the epigenetic regulation of hypoxia response pathways, underscoring the potential therapeutic implications of targeting this axis in diseases characterised by hypoxic stress. The present study explored the interaction between GPX3, Hif1α and LSD1 in the setting of hypoxic injury in vitro and MI in vivo.

In this study, we report a novel discovery that GPX3 levels are significantly elevated in both myocardial infarction tissues and plasma. Using a cardiomyocyte‐specific GPX3 overexpression adenovirus, both in vitro and in vivo experiments demonstrated that GPX3 promotes cardiomyocyte survival and myocardial repair post‐MI. These findings provide new insights and potential therapeutic implications for the use of GPX3 in MI treatment.

## 2 Methods

### 2.1 Development of Mice MI Model

All experimental procedures were approved by the Animal Management and Use Committee and authorised by the Animal Ethics Committee of Nanjing Medical University (No. 2403040), adhering to the Jiangsu Province Experimental Animal Management Measures (Jiangsu Provincial People's Government No. 45). We utilised 8‐week‐old male ICR mice weighing 22–30 g and neonatal rats aged 1–3 days from the Animal Center of Nanjing Medical University. The animals were housed in a specific pathogen‐free (SPF) environment at a controlled temperature of 21°C ± 2°C, humidity of 50% ± 15% and a 12‐h light–dark cycle, with ad libitum access to water and food.

The myocardial infarction (MI) model was induced in the mice using a standardised protocol [30]. Mice were anaesthetised with 1.2% Avertin (40 mg/kg; Sigma‐Aldrich, USA) and ventilated via tracheal intubation. Under a stereomicroscope, the left anterior descending coronary artery (LAD) was located and permanently ligated at the lower end of the left atrial appendage using a 7‐0 suture. The thoracic cavity and skin were closed meticulously with 3‐0 nonabsorbable silk sutures. The sham group underwent the same surgical procedures, except for the LAD ligation. Postoperatively, the mice were maintained on a heat stage until they recovered from anaesthesia. Mice were euthanised at 3 and 28 days post‐MI to collect hearts, with a 2‐mm‐wide myocardium around the infarct area being dissected for subsequent analysis.

### 2.2 Development of Pigs I/R Model

Adult Bama pigs (12 ± 2 months old, weighing 25 ± 5 kg) were acquired from the Animal Center of Nanjing Medical University. These animals were housed in conditions maintained at 22°C –25°C with a 12‐h light–dark cycle. All experimental procedures adhered to established guidelines for animal management and were approved by the Animal Ethics Committee of Nanjing Medical University (IACUC‐2005033).

The ischaemia/reperfusion (I/R) model in adult pigs was developed based on protocols from prior studies [31]. An initial health assessment was performed using echocardiographic measurements obtained via the Simpson method. Anaesthesia was induced with an intramuscular injection of Zoletil50 (4–6 mg/kg) and maintained through tracheal intubation, with a continuous infusion of 1%–2% isoflurane in a 60% oxygen and 40% air mixture using positive‐pressure ventilation. In addition, a glucose–insulin–potassium (GIK) solution was administered intravenously at a rate of 30 drops/min. Vital signs were continuously monitored throughout the procedure. The left anterior descending artery (LAD) was exposed through a mid‐thoracic incision and isolated with a 5‐0 sliding suture. Ischaemic preconditioning was applied for 5 min to reduce the risk of ventricular arrhythmias before completely ligating the LAD. Signs of myocardial discoloration and segmental motion abnormalities were noted during the occlusion period. After maintaining the occlusion for 60 min, reperfusion was initiated by releasing the suture, and the thoracic cavity was immediately closed. The control group underwent a similar thoracotomy without LAD ligation. Arrhythmias were managed with continuous infusions of lidocaine (1.5 mg/kg) and amiodarone (5 mg/kg), supplemented with electrical defibrillation when necessary.

Pigs that exhibited a left ventricular ejection fraction (LVEF) between 30%–45% one hour post‐ligation were selected for follow‐up studies. Seven days post‐surgery, myocardial tissue samples from the infarct border zone were collected for subsequent proteomic analysis.

### 2.3 Neonatal Rat Cardiomyocytes Isolation, Culture and the Oxygen–Glucose Deprivation (OGD) Model Establishment

Neonatal rat cardiomyocytes (NRCMs) were harvested from the hearts of 1‐ to 3‐day‐old Sprague–Dawley rats, using a method previously described [32]. Briefly, ventricular tissues were finely minced and subjected to sequential enzymatic digestion in a solution containing 0.12 mg/mL pancreatin (Sigma, USA) and 0.08 mg/mL collagenase (Worthington, USA). Each digestion cycle lasted 6–8 min until complete dissociation into single cells was achieved. The resultant cell mixture was then filtered through a 100‐μm mesh to remove tissue debris. Cells were cultured in DMEM supplemented with 10% foetal bovine serum (Gibco, 16010159) for approximately 45 min to allow for the adherence of fibroblasts, which were subsequently removed. The remaining cells were further purified using Percoll density gradient centrifugation at 3000 rpm for 30 min, ensuring gradual acceleration and deceleration. The middle layer, enriched with NRCMs, was collected and cultured for 24–36 h prior to experimentation.

An oxygen–glucose deprivation (OGD) model was established to simulate ischaemic conditions. Cultured cells were rinsed with PBS and incubated in serum‐free and glucose‐free DMEM within an AnaeroPACK Rectangular Jar (INC, Japan, Mitsubishi Gas Chemical Company) set to an atmosphere of 1% O_2_, 5% CO_2_ and 94% N_2_ for a duration of 8 h.

### 2.4 Transfection of Recombinant Adenovirus

The CM‐specific recombinant adenoviruses used in this study, including Ad5:cTNT‐GPX3, Ad5:cTNT‐GPX3i, Ad5:cTNT‐LSD1, Ad5:cTNT‐LSD1i, Ad5:cTNT‐Hif1αi and Ad5:cTNT‐CON were purchased from Hanbio Biotechnology Company (Shanghai, China). Cells were transfected using these adenoviruses at a multiplicity of infection (MOI) of 50. After an incubation period of 16 h to allow for viral entry and initial gene expression, the culture medium was replaced with complete medium to support further cellular processes and expression.

### 2.5 Echocardiography Measurement

Echocardiographic assessments were performed using a Visual Sonics Vevo 2100 system (VisualSonics, Canada) equipped with a 40‐MHz high‐resolution mouse ultrasound probe. This noninvasive technique was employed to evaluate cardiac function in mice following MI or sham surgery. At 4 weeks post‐operation, mice were anaesthetised with 0.5%–1.0% isoflurane during the imaging procedure. Cardiac dimensions and function were assessed by acquiring M‐mode and long‐axis view images, from which the left ventricular inner diameters during systole (LVIDs) and diastole (LVIDd) were measured. Left ventricular ejection fraction (EF) and fractional shortening (FS) were then calculated using standard formulas. These measurements were derived from the average values of at least five consecutive cardiac cycles, ensuring accuracy and reproducibility of the data.

### 2.6 Flow Cytometry

NRCMs were seeded at a density of 8 × 10^5^ cells per well in 6‐well plates. The cells were categorised into different groups based on the specific adenoviral interventions employed, with some groups additionally subjected to oxygen–glucose deprivation (OGD) to simulate ischaemic conditions. Following treatment, cells were harvested and prepared for apoptosis analysis according to the protocol provided with the Annexin V‐APC Apoptosis Detection Kit (KGA1101, KeyGEN Biotech). Flow cytometric analysis was performed using a flow cytometer (Tree Star, USA) to quantify apoptotic cells. In this analysis, cells staining positive for Annexin V and negative for propidium iodide (PI) were identified as early apoptotic, whereas cells positive for both Annexin V and PI were classified as late apoptotic.

### 2.7 Histology and Immunohistochemistry Staining

Mouse hearts were harvested, immediately rinsed in cold phosphate‐buffered saline (PBS) and fixed in 4% formaldehyde. Following fixation, the hearts were embedded in paraffin and sectioned at a thickness of 5 μm. These sections included the infarcted myocardium, the border zone and the remote noninfarcted myocardial areas, designated for detailed subsequent analyses. Staining of the sections was performed using Masson's trichrome (Solarbio, G1340) and Sirius red (Abcam, ab150681), following the respective standard protocols. Imaging of the stained sections was carried out with a Nikon Eclipse 50i microscope (Nikon, Japan), and the fibrotic areas were quantitatively assessed using Image Pro Plus software.

### 2.8 Immunofluorescent Staining

NRCMs and myocardial tissues were fixed with 4% paraformaldehyde, permeabilised with 0.2% Triton X‐100 for 15 min and blocked using 10% goat serum. The specimens were incubated with primary antibodies overnight at 4°C and with fluorescent secondary antibodies for 1 h at room temperature to facilitate identification. Nuclear staining was performed using DAPI (Sigma, USA). The primary antibodies utilised were anti‐GPX3 (Abcam, ab256470) and anti‐cTNT (Abcam, ab209813). The secondary antibodies included Alexa Fluor 647 goat anti‐rabbit (Thermo Fisher, A21446) and Alexa Fluor 488 goat anti‐mouse (Thermo Fisher, A11034). Additional staining with DHE, MitoSOX Red and JC‐1 was conducted according to standard protocols under dark conditions. Cell apoptosis was assessed using the TdT mediated dUTP Nick End Labeling (TUNEL) Apoptosis Detection Kit (Yeasen, 40308ES) following the manufacturer's instructions. Images were captured using a Zeiss‐LSM800 rotating disk confocal microscope, and apoptotic cells were quantified by determining the percentage of TUNEL‐positive cells relative to the total cell count, the result of which was expressed as a percentage.

### 2.9 RNA Extraction and Quantitative Real‐Time PCR (qRT‐PCR)

Total RNA was extracted from cultured NRCMs and adult mouse hearts utilising Trizol reagent (Vazyme, R401‐01), adhering to the manufacturer's guidelines. The concentration of RNA was quantified with a NanoDrop‐2000 spectrophotometer (Thermo Fisher Scientific). Subsequent reverse transcription was performed using HiScript III RT SuperMix for cDNA synthesis. Quantitative real‐time PCR (qRT‐PCR) analyses were conducted on a QuantStudio 5 system (ABI) using ChamQ SYBR qPCR Master Mix. This study included at least three biological replicates per experimental group. Primer sequences are detailed in Table S1.

### 2.10 Western Blotting

Total protein was isolated from myocardial tissues and primary cardiomyocytes using RIPA Lysis Buffer (Beyotime, P0028), following the manufacturer's recommended protocol. The antibodies used for immunoblotting included: anti‐GPX3 (Abcam, ab256470), anti‐Bcl‐2 (Abcam, ab196495), anti‐Bax (Cell Signaling Technology, 14796), anti‐Cleaved‐caspase‐3 (Cell Signaling Technology, 9664), anti‐Collagen I (Wanleibio, WL0088), anti‐MMP2 (Proteintech, 10373‐2‐AP), anti‐α‐SMA (Proteintech, 80008‐1‐RR), anti‐LSD1 (Abcam, ab129195), anti‐Hif1α (Cell Signaling Technology, 36169), anti‐β‐actin (Proteintech, 81115‐1‐RR), anti‐GAPDH (Proteintech, 60004‐1‐Ig).

### 2.11 Plasma Sample Collection From AMI Patients

Human plasma samples were obtained from 40 healthy individuals attending routine health screenings and 40 patients diagnosed with AMI, as defined by the Fourth Universal Definition of Myocardial Infarction (2018) [33], at Jiangsu Province Hospital between January 2021 and June 2023. Exclusion criteria included: individuals with a prosthetic heart valve, recent cardiac stent placement or cardiac revascularisation within the past year, myocarditis or uncontrolled severe arrhythmia, aortic aneurysm or carotid artery dissection, severe hepatic and renal diseases, severe haematological diseases or infectious diseases, severe cognitive impairment, autoimmune system diseases, severe respiratory disease, thyroid dysfunction and active cancer. Ethical approval for the study was granted by the local ethics committee (Approval No: 2022‐SR‐646), and informed consent was secured from all participants before their inclusion in the study.

### 2.12 Laboratory Analyses of Glutathione Peroxidase 3 Activity

Human blood samples were collected following an overnight fast and subsequently centrifuged at 1000 rpm for 10 min before being stored at −80°C. The activity of GPX3 in the serum was determined using a coupled enzyme activity assay (Beyotime, S0056). This assay relies on GPX3 catalysing the conversion of reduced glutathione (GSH) to oxidised glutathione (GSSG), followed by the reversion of GSSG to GSH by glutathione reductase, utilising nicotinamide adenine dinucleotide phosphate (NADPH) as a substrate. The GPX3 activity is quantified by monitoring the rate of NADPH consumption, which is reflected by a decrease in absorbance at 340 nm, as mediated by glutathione reductase.

### 2.13 Statistical Analysis

Data were presented as mean ± SEM. Statistical analyses were conducted using GraphPad Prism version 8.0 (GraphPad Software, CA). Continuous variables underwent normality assessment using the Shapiro–Wilk test. For data conforming to a normal distribution, differences between two groups were evaluated using an unpaired two‐sided Student's *t*‐test, while an analysis of variance (ANOVA) with Bonferroni correction was applied for multiple group comparisons. For data that did not adhere to normal distribution, nonparametric tests were utilised. The Mann–Whitney *U* test was implemented for comparisons between two groups, and the Kruskal–Wallis test was used for analyses involving multiple groups. Differences were considered statistically significant at *p* < 0.05.

## 3 Results

### 3.1 GPX3 Expression Was Upregulated After Acute Myocardial Injury

**FIGURE 1:**
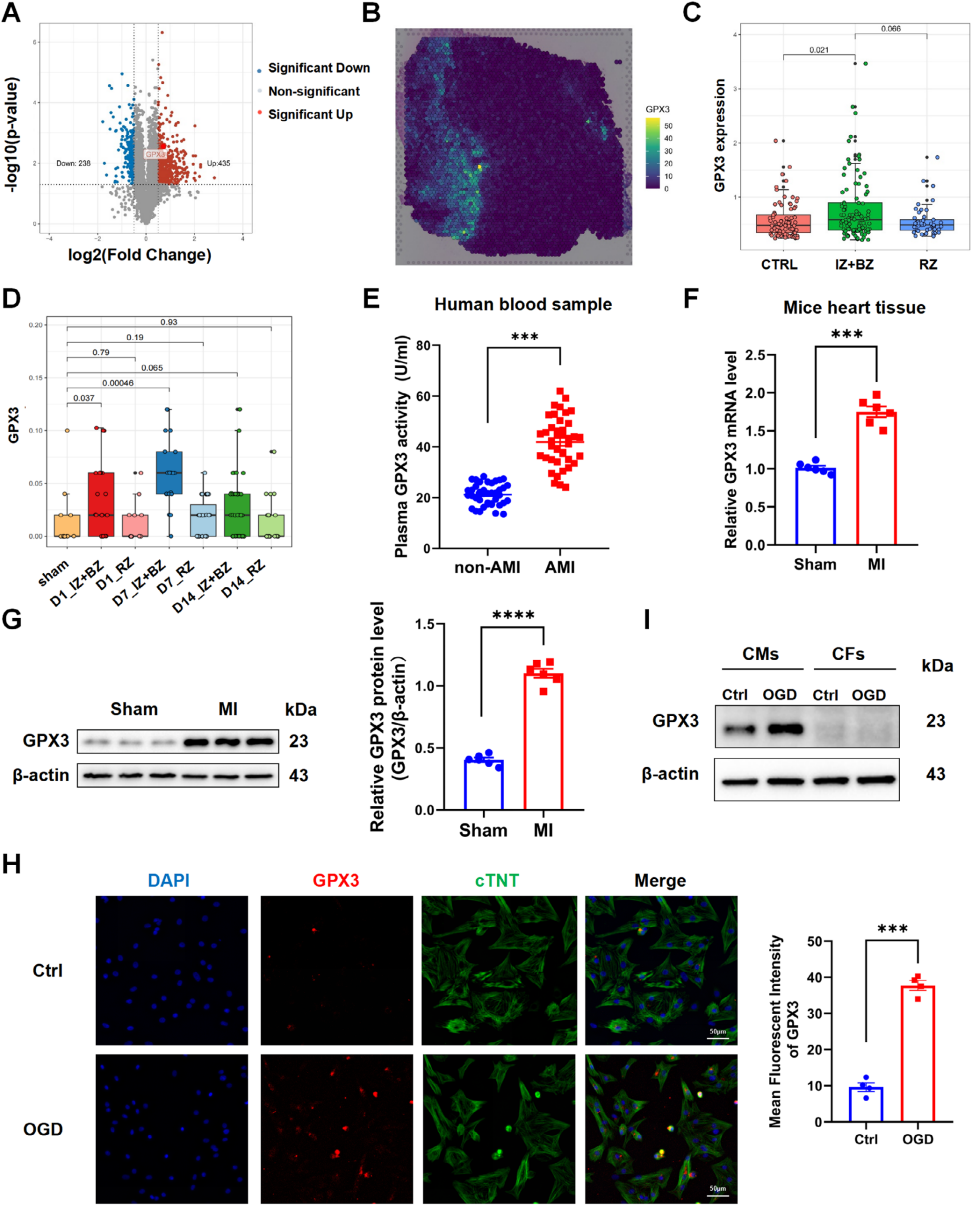
The expression of Glutathione peroxidase 3 (GPX3) was significantly upregulated after acute myocardial infarction (AMI). (A) The volcano plot displays the significant‐down, significant‐up and nonsignificant proteins in normal and ischaemic swine heart tissues. Proteins with fold changes greater than two‐fold and a *p* value less than 0.05 are included. (B) Spatial expression of GPX3 in human heart samples with acute myocardial infarction. (C) Boxplot depicting the expression levels of GPX3 in human cardiomyocytes following MI, categorised into control, ischaemic and border zone (IZ + BZ) and remote zone (RZ). Data obtained from single‐nucleus RNA sequencing (snRNA‐seq). (D) Boxplot depicting the expression levels of GPX3 in mouse cardiomyocytes at 1, 7 and 14 days post‐AMI. Data are categorised into control, ischaemic and border zone (IZ + BZ) and remote zone (RZ) from single‐nucleus RNA sequencing (snRNA‐seq). (E) Measurement of GPX3 activity levels in the plasma of patients with or without AMI within 7 days, using a colorimetric assay kit (*n* = 40). (F) Quantitative real‐time PCR (qRT‐PCR) analysis of GPX3 mRNA levels in cardiac tissues from mice subjected to MI or sham surgery, measured 3 days post‐operation (*n* = 6). (G) Comparison of GPX3 protein expression in mouse hearts post‐MI surgery versus controls (*n* = 6). (H) Representative immunofluorescence images of GPX3 levels in neonatal rat cardiomyocytes following oxygen–glucose deprivation (OGD) treatment (*n* = 3). (I) GPX3 protein expression in neonatal rat cardiomyocytes and fibroblasts with or without OGD treatment. Data are presented as mean ± SEM. ***p < 0.001. **** *p* < 0.0001.

We proposed that GPX3 could be involved in the process of cardiac injury after MI; hence, we initially assessed the expression patterns of GPX3 across multiple species via multi‐omics analyses. In the swine model of acute myocardial ischaemia/reperfusion (I/R), myocardial tissues were collected from the infarct border zone 7 days post‐I/R, as well as from comparable areas in the sham‐operated group. These samples were subjected to mass spectrometry to identify proteins with differential expression (Table S2). Notably, GPX3 expression was significantly elevated in the post‐I/R swine model compared to the sham group (Figure 1A). In addition, we extracted and analysed three single‐nucleus RNA sequencing (snRNA‐seq) datasets of post‐MI myocardial tissue, separately obtained from human and mouse samples, from the GEO public database to assess GPX3 expression in CMs [32, 33, 34]. These analyses revealed increased GPX3 expression in CMs within the ischaemic zone (IZ) and border zone (BZ) of infarcted hearts in both humans and mice (Figure 1B–D). Furthermore, plasma GPX3 activity was elevated in AMI patients compared to healthy controls (Figure 1E). Experimental mouse MI models also demonstrated that both mRNA and protein levels of GPX3 were significantly increased post‐AMI (Figure 1F,G). In vitro, we constructed hypoxia models in CM and fibroblasts, and found that GPX3 was predominantly expressed in CMs and exhibited significant upregulation following hypoxic conditions (Figure 1H,I). These results indicated that GPX3 may be involved in the myocardial injury and repair process following MI.

### 3.2 GPX3 Overexpression Ameliorated Oxygen–Glucose Deprivation (OGD) Induced Oxidative Damage and Apoptosis In Vitro

**FIGURE 2:**
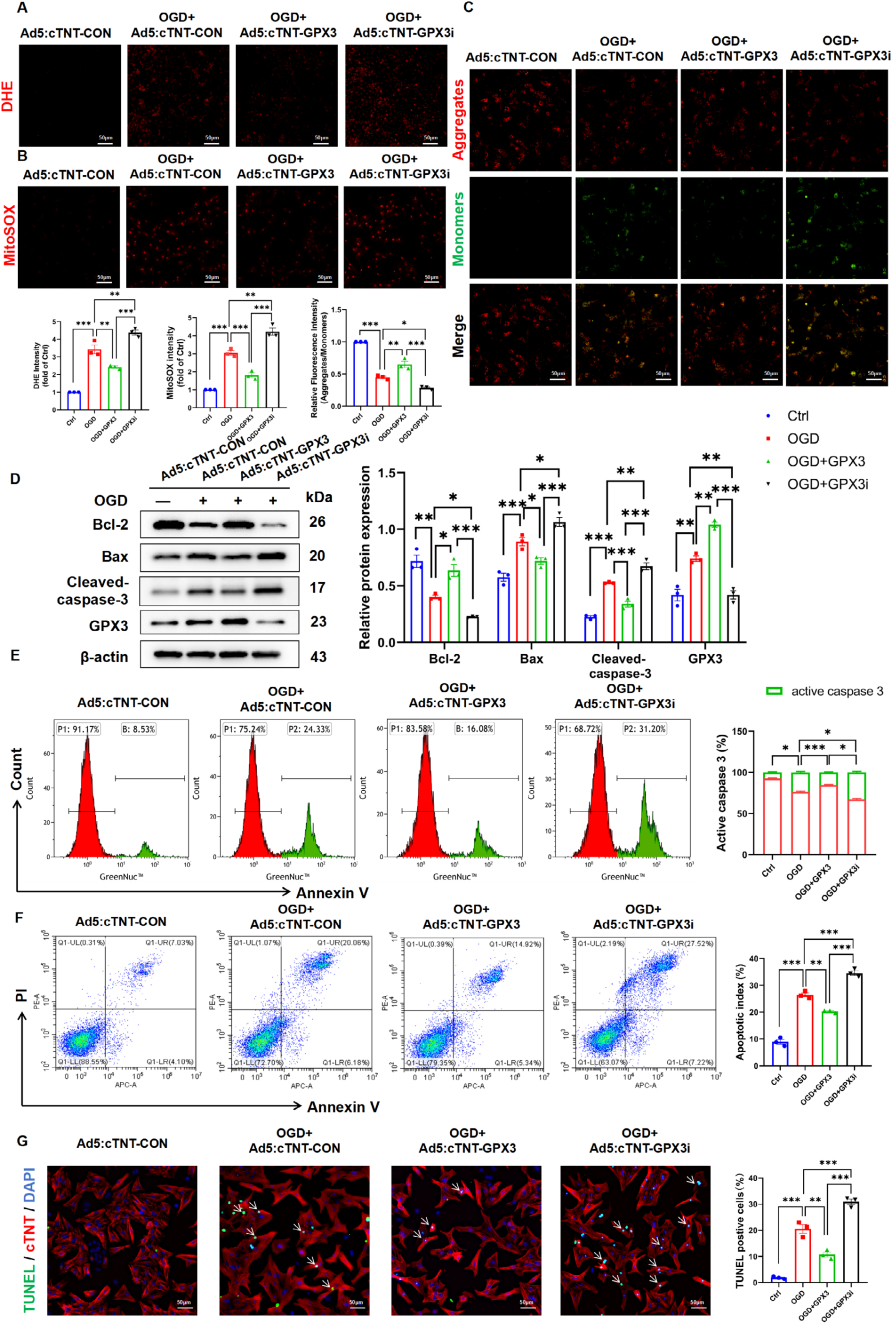
Effects of GPX3 (Glutathione peroxidase 3) on oxidative stress and apoptosis in NRCMs. (A) Representative confocal microscope images and quantitative analysis of reactive oxygen species (ROS) levels after DHE staining in neonatal rat cardiomyocytes (NRCMs) with GPX3 overexpression or knockdown (*n* = 3). (B) Representative confocal microscope images and quantitative analysis of mitochondrial ROS using MitoSOX Red staining in NRCMs across four experimental groups: Control, OGD, OGD + GPX3 and OGD + GPX3i (*n* = 3). (C) JC‐1 staining and quantitative analysis to detect changes in mitochondrial membrane potential (MMP) in CMs treated differently (*n* = 3). (D) Western blot analysis showing the protein levels of Bcl‐2, Bax, Cleaved‐caspase‐3 and GPX3 in CMs under various treatments (*n* = 3). (E) Flow cytometry analysis of caspase‐3 activity in CMs (*n* = 3). (F) Flow cytometry assessment of early and late apoptotic cells in CMs (*n* = 3). (G) TUNEL staining of CMs subjected to OGD conditions (*n* = 3). Data are presented as mean ± SEM. Significance levels are indicated as follows: *p < 0.05, **p < 0.01, ***p < 0.001.

To explore the potential effect of GPX3 against hypoxia‐induced injury in CMs, NRCMs were transfected with Ad5:cTNT‐GPX3, Ad5:cTNT‐GPX3i and corresponding control adenoviruses for 48 h prior to oxygen–glucose deprivation (OGD) treatment. As an antioxidant enzyme, GPX3 mainly functions to eliminate superoxide radicals, which leads us to question whether GPX3 regulates oxidative stress levels in CMs [6]. For this purpose, we perform DHE staining and the results confirmed the reactive oxygen species (ROS) scavenging efficacy of GPX3 (Figure 2A). Mitochondrial function and membrane permeability were assessed using MitoSOX Red and JC‐1 staining, respectively. Results showed that mitochondrial ROS decreased with GPX3 overexpression, whereas GPX3 deficiency exacerbated mitochondrial ROS (Figure 2B). Additionally, JC‐1 staining showed that GPX3 depletion reduced mitochondrial membrane potential (MMP), while overexpression of GPX3 restored MMP levels (Figure 2C). Western blot analysis further corroborated the anti‐apoptotic effects of GPX3 in NRCMs exposed to OGD. Specifically, there was an upregulation of the anti‐apoptotic protein Bcl‐2 and a downregulation of the proapoptotic proteins Bax and Cleaved‐caspase‐3 in the Ad5:cTNT‐GPX3 treated group. In contrast, GPX3 knockdown resulted in elevated levels of Bax and Cleaved‐caspase‐3 in CMs (Figure 2D). Flow cytometry analysis also indicated reduced caspase‐3 activity following GPX3 overexpression (Figure 2E). TUNEL staining and flow cytometry demonstrated a significant reduction in the apoptosis rate of CMs in the OGD + GPX3 group compared to the OGD control groups, and the GPX3 knockdown group exhibited the highest apoptosis rate (Figure 2F,G). Collectively, these results suggested that GPX3 can maintain mitochondrial redox balance in CMs and protect against OGD‐induced apoptosis.

### 3.3 GPX3 Overexpression Ameliorated Cardiac Injury Post‐MI In Vivo

**FIGURE 3:**
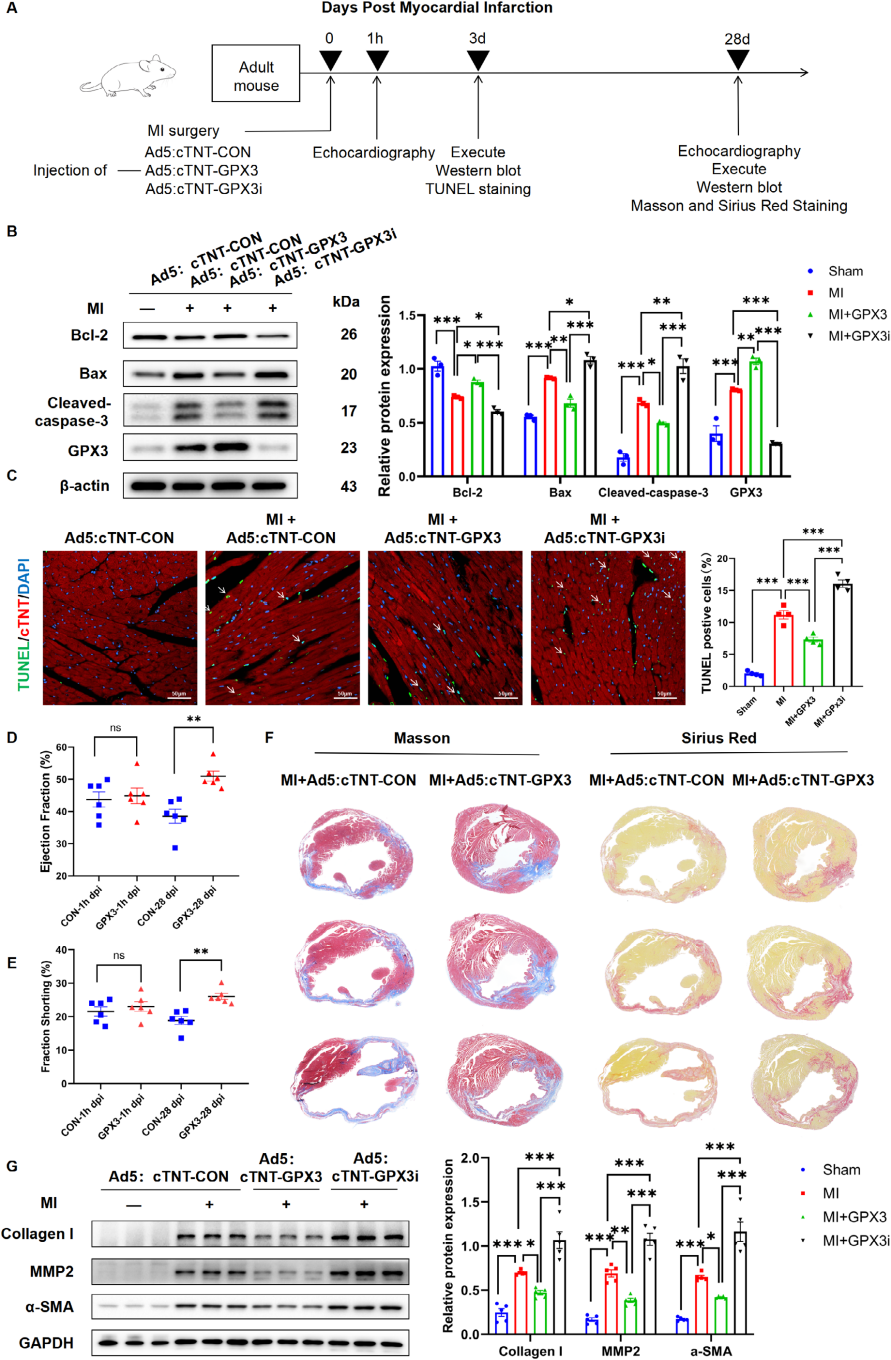
GPX3 (Glutathione peroxidase 3) overexpression reduced cardiomyocyte loss and preserved cardiac function in mice 28 days post‐MI in vivo. (A) Experimental protocol: Adenovirus 5 (Ad5) was injected into the myocardium at the infarct border immediately after ligation of the left anterior descending artery. Apoptosis rates and relative protein levels were assessed on day 3 post‐MI. Echocardiography was performed on day 28 post‐infarction to evaluate cardiac function, followed by the harvesting of hearts for further analysis. (B) Western blot analysis of Bcl‐2, Bax, Cleaved‐caspase‐3 and GPX3 protein levels in mouse hearts (*n* = 3). (C) Representative images and quantitative data of TUNEL staining among different groups (*n* = 4). (D–E) Echocardiographic measurements of left ventricular ejection fraction (LVEF) and left ventricular fractional shortening (LVFS) among the different groups (*n* = 6). (F) Representative images and quantification of Masson's trichrome and Sirius red staining at 28 days following MI or sham surgery (*n* = 5). (G) Protein levels and quantitative analysis of Collagen I, MMP2 and α‐SMA in heart tissue post‐surgery (*n* = 5). Data are presented as mean ± SEM. Significance levels are indicated as follows: *p < 0.05, **p < 0.01, ***p < 0.001.

To further elucidate the protective role of GPX3 on ischaemic myocardium during the acute stage of MI in adult mice, Ad5:cTNT‐CON, Ad5:cTNT‐GPX3 or Ad5:cTNT‐GPX3i were administered locally immediately after LAD artery ligation in P56 mouse hearts (Figure 3A). Western blot analysis confirmed the overexpression of GPX3 in the MI + Ad5:cTNT‐GPX3 group and a decrease in GPX3 expression in the MI + Ad5:cTNT‐GPX3i group. Furthermore, overexpression of GPX3 significantly enhanced the levels of the anti‐apoptotic protein Bcl‐2, while reducing the levels of the proapoptotic proteins Cleaved‐caspase‐3 and Bax. Additionally, it decreased the proportion of apoptotic CMs in the border zone of the infarct, as evidenced by TUNEL staining. Conversely, the GPX3 knockdown group displayed the opposite effects (Figure 3B,C). These findings support the beneficial role of GPX3 in reducing apoptosis post‐MI in vivo.

To further explore the effects of GPX3 overexpression on adverse ventricular remodelling and cardiac function recovery after MI, relevant experiments were conducted 28 days post‐MI. Echocardiographic evaluation revealed that GPX3 overexpression significantly improved cardiac function, as indicated by enhanced left ventricular ejection fraction (LVEF) and left ventricular fractional shortening (LVFS). Specifically, the cardiac function index of EF (50.97% ± 1.40%) and FS (26.06% ± 0.84%) was significantly higher in the GPX3 overexpression group compared to the control group (EF:38.53% ± 2.02%, *p* = 0.003; FS: 18.86% ± 1.10%, *p* = 0.0029) (Figure 3D,E). Histological analysis, performed using Masson's trichrome and Sirius red staining techniques, revealed a reduced level of collagen deposition and fibrosis in the infarcted myocardium of mice with GPX3‐overexpression, compared to the control group (Figure 3F). Molecular analysis by Western blot further supported these findings, showing a significant decrease in the expression of fibrosis‐related proteins such as α‐SMA, MMP2 and Collagen I in the GPX3 overexpression group relative to controls (Figure 3G).

### 3.4 Higher Hif1α Expression Is Essential for the Anti‐Apoptotic Effect of GPX3 on CMs

**FIGURE 4:**
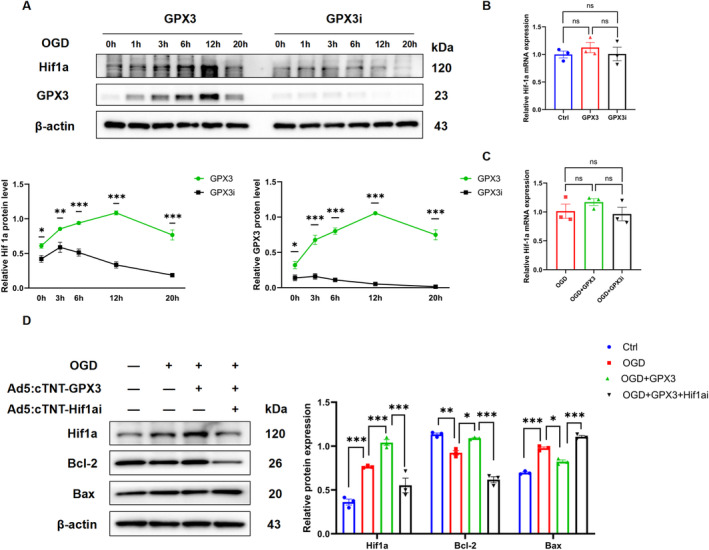
Glutathione peroxidase 3 (GPX3) overexpression promotes Hif1α to mitigate apoptosis. (A) Western blot analysis of Hif1α and GPX3 protein levels in CMs with GPX3 overexpression or knockdown, measured under hypoxic conditions at various time points (*n* = 3). (B) Hif1α mRNA levels in CMs assessed under normoxic conditions following various treatments (*n* = 3). (C) Hif1α mRNA levels in CMs measured under hypoxic conditions after different treatments (*n* = 3). (D) Western blot analysis of Hif1α, Bcl‐2 and Bax protein levels in OGD‐induced CMs transfected with either Ad5:cTNT‐GPX3, Ad5:cTNT‐Hif1αi or both (*n* = 3). Data are presented as mean ± SEM. Significance levels are indicated as follows: *p < 0.05, **p < 0.01, ***p < 0.001.

Further, we investigated the mechanism by which GPX3 influences MI damage and apoptosis. Referring to relevant literature [34], GPX3 is posited to affect the expression of the Hif1α protein. Moreover, it is well‐known that under hypoxic conditions, Hif1α can dimerise with Hif1β to form the Hif1 complex, which in turn regulates the expression of downstream genes critical for maintaining oxygen homeostasis and ensuring cell survival. To explore whether GPX3 modulates Hif1α expression levels and thereby affects the cellular response to ischemic stress and apoptosis in myocardial cells, we first intervened GPX3 expression by Ad5:cTNT‐GPX3 and Ad5:cTNT‐GPX3i in NRCMs under hypoxic conditions. Western blot analysis revealed that overexpression of GPX3 could increase Hif1α protein expression, whereas knockdown of GPX3 diminished Hif1α expression under hypoxic conditions (Figure 4A). However, neither overexpression nor knockdown of GPX3 had a significant effect on Hif1α mRNA expression under either normoxic or hypoxic conditions, revealing that this change was likely to be regulated post‐transcriptionally (Figure 4B,C). We also transfected Ad5:cTNT‐GPX3 with or without Ad5:cTNT‐Hif1αi in CMs. Western blot analysis showed that knockdown of Hif1α abrogated the anti‐apoptotic effects facilitated by GPX3, confirming the critical role of Hif1α in the protective mechanism of GPX3 in cardiomyocytes (Figure 4D).

### 3.5 GPX3 Overexpression Enhanced Hif1α Expression by Activating LSD1

**FIGURE 5:**
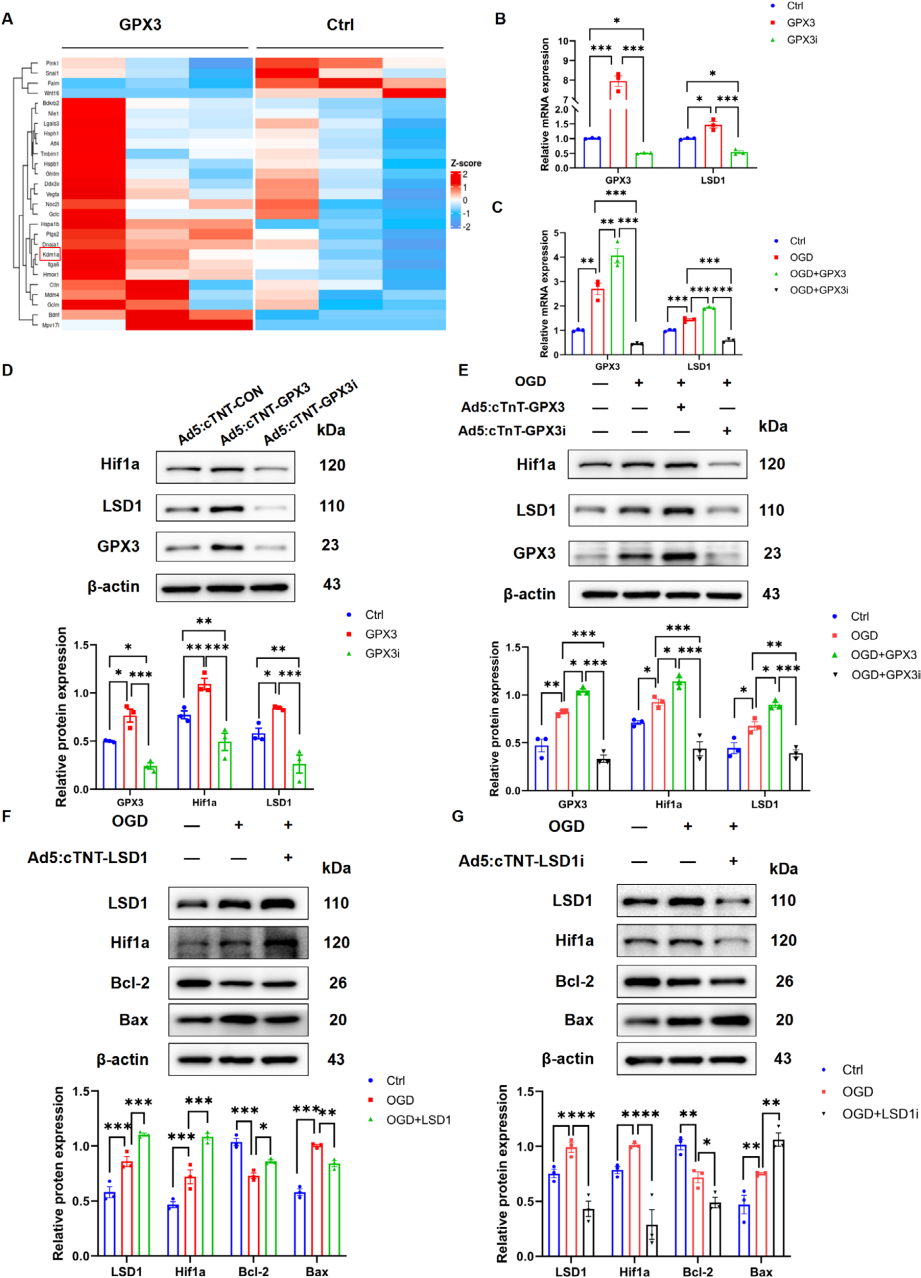
Glutathione peroxidase 3 (GPX3) overexpression enhances Hif1α expression via activation of LSD1. (A) A heatmap displaying protein variations in cardiomyocytes treated with GPX3 or a control. (B, C) The mRNA levels of LSD1 and GPX3 in control, GPX3 overexpression and GPX3 inhibition groups (*n* = 3). (D) Protein levels and quantification of Hif1α, LSD1 and GPX3 in CMs with GPX3 overexpression or knockdown under normoxic conditions (*n* = 3). (E) Protein levels and quantification of Hif1α, LSD1 and GPX3 in CMs with GPX3 overexpression or knockdown under hypoxic conditions (*n* = 3). (F) Protein levels and quantification of LSD1, Hif1α, Bcl‐2 and Bax in OGD‐induced CMs with or without LSD1 overexpression (*n* = 3). (G) Protein levels and quantification of LSD1, Hif1α, Bcl‐2 and Bax in OGD‐induced NRCMs with or without LSD1 knockdown (*n* = 3). Data are presented as mean ± SEM. Significance levels are indicated as follows: *p < 0.05, **p < 0.01, ***p < 0.001.

To elucidate the mechanism by which GPX3 post‐transcriptionally regulate Hif1α, NRCMs were transfected with Ad5:cTNT‐CON and Ad5:cTNT‐GPX3 and RNA‐seq analysis was performed to compare gene expression profiles. A false discovery rate (FDR) corrected *p* value (FDRq) < 0.05 and a |log2(fold change)| > 1 were criteria used to identify differentially expressed genes (DEGs). Among the 597 differentially expressed genes between the two groups, 209 were upregulated and 387 were downregulated in the GPX3 overexpression group. Upon further examination, we observed a significant upregulation of lysine‐specific demethylase 1 (KDM1A/LSD1). This demethylase is known for promoting cell survival and enhancing the stability of Hif1α protein post‐translationally, findings that caught our attention (Figure 5A). We further investigate whether GPX3 could promote Hif1α expression through LSD1. We analysed LSD1 mRNA levels, which were increased in GPX3‐overexpressing groups and decreased in GPX3 knockdown groups under both normoxic and hypoxic conditions (Figure 5B,C). Parallel changes were observed in LSD1 protein levels, which increased with GPX3 overexpression and decreased upon its knockdown; these changes were also accompanied by similar alterations in Hif1α protein expression (Figure 5D,E).

Furthermore, by overexpressing and knocking down LSD1 in CMs, we found that LSD1 modulates Hif1α protein levels and affects apoptosis under hypoxic conditions. Specifically, LSD1 overexpression increased intracellular Hif1α protein and decreased the apoptosis‐related indicator Bax, while increasing the anti‐apoptosis indicator Bcl‐2. Conversely, LSD1 knockdown yielded opposite results (Figure 5F,G). Above all, these results indicated that LSD1 may play a mediating role in the GPX3‐Hif1α axis.

### 3.6 GPX3 Overexpression Attenuated Apoptosis Through GPX3/LSD1/Hif1α Pathway

**FIGURE 6:**
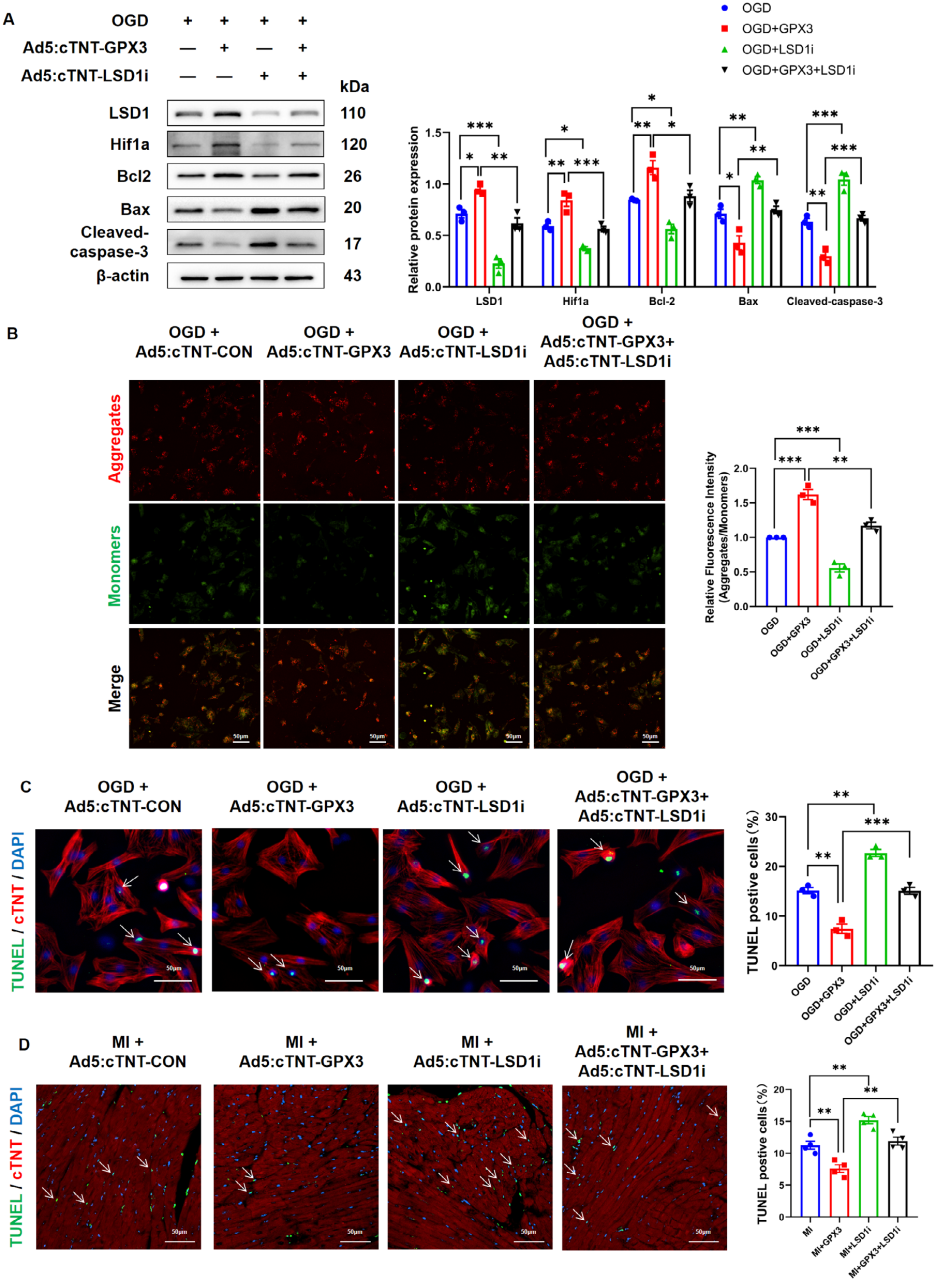
Glutathione peroxidase 3 (GPX3) attenuated the loss of cardiomyocytes via the GPX3/LSD1/Hif1α pathway. (A) Protein quantification and levels of LSD1, Hif1α, Bcl‐2, Bax and Cleaved‐caspase‐3 in CMs subjected to OGD and transfected with Ad5:cTNT‐GPX3, Ad5:cTNT‐LSD1i or both (*n* = 3). (B) Representative confocal microscope images and quantitative analysis of JC‐1 assays detecting MMP in CMs treated with OGD, OGD + GPX3, OGD + LSD1i and OGD + GPX3 + LSD1i (*n* = 3). (C, D) Representative images and quantitative analysis of TUNEL staining in NRCMs subjected to OGD (*n* = 3) and in mouse hearts three days post‐MI (*n* = 4) following various interventions. Data are presented as mean ± SEM. Significance levels are indicated as follows: *p < 0.05, **p < 0.01, ***p < 0.001.

To further elucidate the regulatory role of LSD1 in this process, cultured CMs were treated separately or together with Ad5:cTNT‐GPX3 and Ad5:cTNT‐LSD1i for 48 h, followed by exposure to oxygen–glucose deprivation. Western blot analysis showed that downregulation of LSD1 reversed the anti‐apoptosis effects of overexpressing GPX3, manifested as the decreased Hif1α and Bcl2 levels and upregulated proapoptotic proteins Bax and Cleaved‐caspase‐3 (Figure 6A). Furthermore, JC‐1 staining assays demonstrated a reduction in MMP upon LSD1 knockdown, disrupting the MMP stabilisation observed with GPX3 overexpression, thus indicating an augmentation in early apoptotic cells (Figure 6B). TUNEL staining analysis corroborated these findings, showing that GPX3 overexpression significantly inhibited CM apoptosis, whereas simultaneous knockdown of LSD1 attenuated this protective effect in both in vivo and in vitro settings (Figure 6C,D). Collectively, these findings suggested that GPX3 mediates Hif1α expression through LSD1, thereby enhancing cardiomyocyte survival and facilitating cardiac repair post‐myocardial infarction.

## 4 Discussion

**FIGURE 7:**
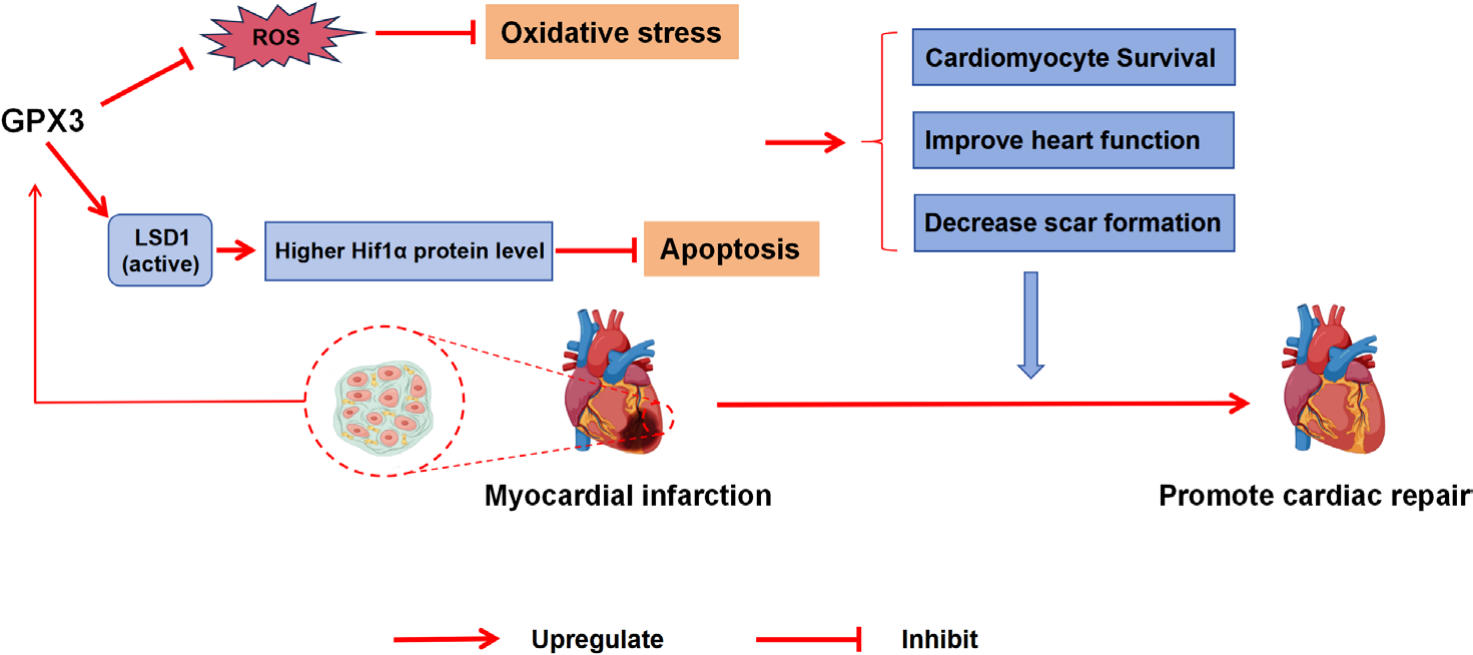
Summary of GPX3 (Glutathione peroxidase 3) on cardiac MI injury. In myocardial tissues subjected to hypoxic conditions following a myocardial infarction, GPX3 expression is significantly upregulated. This increase in GPX3 is associated with a reduction in oxidative stress and apoptosis, which collectively contribute to improved cardiac function. The protective role of GPX3 is partly mediated through its interaction within the GPX3/LSD1/Hif1α axis, enhancing the cellular resilience to hypoxic stress.

In this study, we identified that GPX3, which exhibits increased expression levels in both ischaemic myocardium and serum samples following MI, plays a crucial role in mitigating oxidative stress, reducing CM apoptosis and lessening cardiac injury under hypoxic conditions. Along with CM survival, GPX3 inhibited adverse ventricular remodelling and myocardial fibrosis, and promote cardiac repair and functional recovery. Mechanistically, overexpression of GPX3 significantly upregulated the protein expression of LSD1 and Hif1α, and further rescue experiments showed that GPX3 could play a protective role in ischemic myocardium post‐MI, at least partly, through the LSD1/Hif1α pathway (Figure 7).

GPX3 is widely expressed across various organisms and exhibits variable expression levels under different pathological conditions, correlating with the development and prognosis of numerous benign and malignant diseases [8, 9, 12, 35, 36]. To date, the majority of studies investigating the role of GPX3 in the cardiovascular system have predominantly relied on clinical data, with a particular focus on vascular diseases. Notably, an age‐related decline in GPX3 expression may heighten susceptibility to cardiovascular events in patients with atrial fibrillation [13]. Moreover, a reduction in GPX3 levels has been implicated in the pathogenesis of atherosclerosis and thrombosis [4, 37]. In our study, using a model of MI, we observed an upregulation of GPX3 mRNA and protein levels in cardiomyocytes located at the infarct border zone, suggesting an involvement of GPX3 in both cardiac injury and repair processes. Although GPX3 is typically secreted extracellularly to function as a peroxidase, our findings provide novel evidence of its crucial role in mitigating intracellular reactive oxygen species (ROS) damage and maintaining mitochondrial function within cardiomyocytes. We demonstrated that in vitro overexpression of GPX3 could attenuate ROS production and preserve mitochondrial membrane balance. Furthermore, GPX3 overexpression in cardiomyocytes significantly reduced the proportion of cells undergoing both early and late apoptosis. The dual protective effects of GPX3, encompassing antioxidant and anti‐apoptotic actions during the early phase of MI, could facilitate cellular survival under adverse conditions, mitigate disruption of microenvironmental homeostasis, and thereby prevent negative outcomes associated with MI [38]. Additionally, our findings elucidate the sustained and robust protective influence of GPX3 throughout the reparative phase of MI, characterised by reduced scar formation and improved cardiac function. Consistent with observations from other pathologies, GPX3 has also been shown to diminish fibrosis in conditions such as hepatocellular carcinoma invasiveness [7] and is emerging as a potential therapeutic target for treating fibrosis associated with interstitial lung disease, chronic kidney disease and various cardiac disorders [10, 39, 40, 41]. Conclusively, these multifaceted protective properties of GPX3 enhance cardiac repair and functional recovery after myocardial infarction, further providing valuable insights for developing novel therapeutic strategies in clinical settings.

Hif1α is crucial in regulating processes essential for cardiac growth, development and disease pathology [15, 42, 43]. Previous research has highlighted its significant cardioprotective effects in ischemic heart disease, including inhibiting cardiac fibroblast proliferation, reducing oxidative stress, promoting neovascularisation and decreasing cardiomyocyte apoptosis [30, 44, 45]. The stability of the Hif1α protein, which undergoes various post‐translational modifications, is vital for its transcriptional function and the cellular response to hypoxia, thereby playing a central role in cardioprotection. Factors such as CREB, LRP5 and Hmox1 have been identified as crucial in maintaining Hif1α stability within the cardiovascular system, thus facilitating tissue repair and recovery [22, 46, 47, 48]. In alignment with this, Wang et al. reported that GPX3 impedes Hif1α degradation, thereby promoting its accumulation in MLE‐12 cells, a mouse lung epithelial cell line [34]. Consistent with previous studies, our findings demonstrate that GPX3 modulates Hif1α protein expression in cardiomyocytes. We observed that knockdown of Hif1α significantly diminishes the cardioprotective effects of GPX3, highlighting the dependency of its protective action on the presence of stable Hif1α. Moreover, elevated levels of GPX3 and Hif1α proteins are associated with an enhanced anti‐apoptotic response in cardiomyocytes under hypoxic conditions. These findings collectively indicate that the synergistic interaction between GPX3 and Hif1α plays a pivotal role in mediating the cardioprotective effects observed under hypoxic conditions.

In our investigation, RNA‐seq analysis was employed to delineate the alterations in gene expression modulated by GPX3. This analysis indicated that GPX3 overexpression significantly upregulated genes linked to cell survival, including LSD1. Notably, LSD1 has been implicated in the stabilisation of Hif1α protein under hypoxic conditions via several mechanisms, such as inhibiting methylation‐mediated degradation and suppressing hydroxylation‐driven degradation [25, 26, 27]. Based on these observations, we hypothesised that GPX3 might influence Hif1α expression by modulating LSD1 levels. To test this hypothesis, subsequent experimental interventions were conducted. Knockdown of LSD1 resulted in decreased levels of Hif1α and a substantial increase in apoptosis. Additionally, this specific intervention of LSD1 knockdown within the regulatory pathway significantly attenuated both the elevated Hif1α expression and the anti‐apoptotic effects induced by GPX3 overexpression, which highlights the crucial role of LSD1 in modulating these key protective mechanisms. These results confirm the critical role of the GPX3/LSD1/Hif1α pathway in regulating cellular apoptosis and underscore the synergistic interaction among GPX3, Hif1α and LSD1. This synergy enhances the endogenous protective mechanisms in cardiomyocytes and myocardial tissue, particularly under hypoxic or ischaemic conditions. Such interactions are instrumental in reducing ROS production and apoptosis, thereby improving outcomes related to cardiac remodelling. Moreover, given that GPX3 expression tends to decline with age and the onset of an unhealthy lifestyle [13, 49], this reduction may contribute to the observed poorer prognosis following myocardial infarction in older individuals. Therefore, elucidating the regulatory functions of GPX3, particularly in its interactions with LSD1 and Hif1α becomes more meaningful.

Some limitations exist in our study. First, the reparative process following MI encompasses various physiological mechanisms, including inflammation and angiogenesis. However, the specific contributions of GPX3 to these critical pathological processes were not addressed in our study. Second, while we posited that LSD1 plays a pivotal role in the mechanism through which GPX3 promotes Hif1α protein, the exact molecular interactions and pathways involved remain to be fully elucidated.

In conclusion, our experimental findings reveal that GPX3 expression was increased following MI. GPX3 elevation plays a critical role in cardioprotection by effectively scavenging reactive oxygen species (ROS), reducing apoptosis in cardiomyocytes and improving overall cardiac function and remodelling. Overexpressing GPX3 might provide a promising therapeutic strategy for the treatment of AMI.

## Author Contributions

**Qi‐Qi Jiang:** data curation (equal), writing – original draft (equal). **Chong Du:** data curation (equal), visualization (equal). **Ling‐Ling Qian:** data curation (equal), validation (equal). **Tian‐Kai Shan:** formal analysis (equal), visualization (equal). **Yu‐Lin Bao:** methodology (equal), software (equal). **Ling‐Feng Gu:** methodology (equal), resources (equal). **Si‐Bo Wang:** methodology (equal), software (equal). **Tong‐Tong Yang:** investigation (equal), visualization (equal). **Liu‐Hua Zhou:** formal analysis (equal), validation (equal). **Ze‐Mu Wang:** methodology (equal), validation (equal). **Ye He:** formal analysis (equal), resources (equal). **Qi‐Ming Wang:** methodology (equal), supervision (equal). **Hao Wang:** funding acquisition (equal), supervision (equal). **Ru‐Xing Wang:** funding acquisition (equal), supervision (equal). **Lian‐Sheng Wang:** project administration (equal), supervision (equal).

## Conflicts of Interest

The authors declare no conflicts of interest.

## Supporting information


Supplementary Tables 1‐2.

## Data Availability

The data that support the findings of this study are available from the corresponding author upon reasonable request.

## References

[1] M. Walli‐Attaei , P. Joseph , A. Rosengren , et al., “Variations Between Women and Men in Risk Factors, Treatments, Cardiovascular Disease Incidence, and Death in 27 High‐Income, Middle‐Income, and Low‐Income Countries (PURE): A Prospective Cohort Study,” Lancet 396, no. 10244 (2020): 97–109. 32445693 10.1016/S0140-6736(20)30543-2

[2] G. Heusch , I. Andreadou , R. Bell , et al., “Health Position Paper and Redox Perspectives on Reactive Oxygen Species as Signals and Targets of Cardioprotection,” Redox Biology 67 (2023): 102894. 37839355 10.1016/j.redox.2023.102894 PMC10590874

[3] G. Takebe , J. Yarimizu , Y. Saito , et al., “A Comparative Study on the Hydroperoxide and Thiol Specificity of the Glutathione Peroxidase Family and Selenoprotein P,” Journal of Biological Chemistry 277, no. 43 (2002): 41254–41258. 12185074 10.1074/jbc.M202773200

[4] R. C. Jin , C. E. Mahoney , L. Anderson , et al., “Glutathione Peroxidase‐3 Deficiency Promotes Platelet‐Dependent Thrombosis In Vivo,” Circulation 123, no. 18 (2011): 1963–1973. 21518981 10.1161/CIRCULATIONAHA.110.000034 PMC3107543

[5] S. Nirgude and B. Choudhary , “Insights Into the Role of GPX3, a Highly Efficient Plasma Antioxidant, in Cancer,” Biochemical Pharmacology 184 (2021): 114365. 33310051 10.1016/j.bcp.2020.114365

[6] C. Chang , B. L. Worley , R. Phaëton , and N. Hempel , “Extracellular Glutathione Peroxidase GPx3 and Its Role in Cancer,” Cancers 12, no. 8 (2020): 2197. 32781581 10.3390/cancers12082197 PMC7464599

[7] X. Qi , K. T.‐P. Ng , Y. Shao , et al., “The Clinical Significance and Potential Therapeutic Role of GPx3 in Tumor Recurrence After Liver Transplantation,” Theranostics 6, no. 11 (2016): 1934–1946. 27570561 10.7150/thno.16023 PMC4997247

[8] W. Lou , B. Ding , S. Wang , and P. Fu , “Overexpression of GPX3, a Potential Biomarker for Diagnosis and Prognosis of Breast Cancer, Inhibits Progression of Breast Cancer Cells In Vitro,” Cancer Cell International 20, no. 1 (2020): 378. 32782436 10.1186/s12935-020-01466-7 PMC7412804

[9] W. Manzanares , A. Biestro , F. Galusso , et al., “Serum Selenium and Glutathione Peroxidase‐3 Activity: Biomarkers of Systemic Inflammation in the Critically Ill?,” Intensive Care Medicine 35, no. 5 (2009): 882–889. 19034425 10.1007/s00134-008-1356-5

[10] L. Li , M. He , X. Tang , et al., “Proteomic Landscape of the Extracellular Matrix in the Fibrotic Kidney,” Kidney International 103, no. 6 (2023): 1063–1076. 36805449 10.1016/j.kint.2023.01.021

[11] H. Lee , T. Ismail , Y. Kim , et al., “Xenopus gpx3 Mediates Posterior Development by Regulating Cell Death During Embryogenesis,” Antioxidants 9, no. 12 (2020): 1265. 33322741 10.3390/antiox9121265 PMC7764483

[12] K. Demircan , Y. Bengtsson , Q. Sun , et al., “Serum Selenium, Selenoprotein P and Glutathione Peroxidase 3 as Predictors of Mortality and Recurrence Following Breast Cancer Diagnosis: A Multicentre Cohort Study,” Redox Biology 47 (2021): 102145. 34563873 10.1016/j.redox.2021.102145 PMC8476451

[13] D. Pastori , P. Pignatelli , A. Farcomeni , et al., “Aging‐Related Decline of Glutathione Peroxidase 3 and Risk of Cardiovascular Events in Patients With Atrial Fibrillation,” Journal of the American Heart Association 5, no. 9 (2016): e003682. 27609361 10.1161/JAHA.116.003682 PMC5079030

[14] B. L. Worley , Y. S. Kim , J. Mardini , et al., “GPx3 Supports Ovarian Cancer Progression by Manipulating the Extracellular Redox Environment,” Redox Biology 25 (2019): 101051. 30509602 10.1016/j.redox.2018.11.009 PMC6859581

[15] N. Chi , “Molecular Determinants of Responses to Myocardial Ischemia/Reperfusion Injury: Focus on Hypoxia‐Inducible and Heat Shock Factors,” Cardiovascular Research 61, no. 3 (2004): 437–447. 14962475 10.1016/j.cardiores.2003.11.033

[16] T. Schmid , J. Zhou , and B. Brüne , “HIF‐1 and p53: Communication of Transcription Factors Under Hypoxia,” Journal of Cellular and Molecular Medicine 8, no. 4 (2004): 423–431. 15601571 10.1111/j.1582-4934.2004.tb00467.x PMC6740063

[17] A. Greijer , P. Van Der Groep , D. Kemming , et al., “Up‐Regulation of Gene Expression by Hypoxia Is Mediated Predominantly by Hypoxia‐Inducible Factor 1 (HIF‐1),” Journal of Pathology 206, no. 3 (2005): 291–304. 15906272 10.1002/path.1778

[18] X. Li , Q. Zhang , M. Nasser , et al., “Oxygen Homeostasis and Cardiovascular Disease: A Role for HIF?,” Biomedicine & Pharmacotherapy 128 (2020): 110338. 32526454 10.1016/j.biopha.2020.110338

[19] G. D'Angelo , E. Duplan , N. Boyer , P. Vigne , and C. Frelin , “Hypoxia Up‐Regulates Prolyl Hydroxylase Activity,” Journal of Biological Chemistry 278, no. 40 (2003): 38183–38187. 12876291 10.1074/jbc.M302244200

[20] J. Cheng , X. Kang , S. Zhang , and E. T. H. Yeh , “SUMO‐Specific Protease 1 Is Essential for Stabilization of HIF1alpha During Hypoxia,” Cell 131, no. 3 (2007): 584–595. 17981124 10.1016/j.cell.2007.08.045 PMC2128732

[21] G. L. Semenza , “HIF‐1, O2, and the 3 PHDs,” Cell 107, no. 1 (2001): 1–3. 11595178 10.1016/s0092-8674(01)00518-9

[22] X. Wei , S. Chen , T. Xie , et al., “An MMP‐Degradable and Conductive Hydrogel to Stabilize HIF‐1α for Recovering Cardiac Functions,” Theranostics 12, no. 1 (2022): 127–142. 34987638 10.7150/thno.63481 PMC8690911

[23] Y. Shi , F. Lan , C. Matson , et al., “Histone Demethylation Mediated by the Nuclear Amine Oxidase Homolog LSD1,” Cell 119, no. 7 (2004): 941–953. 15620353 10.1016/j.cell.2004.12.012

[24] F. Gu , Y. Lin , Z. Wang , et al., “Biological Roles of LSD1 Beyond Its Demethylase Activity,” Cellular and Molecular Life Sciences 77, no. 17 (2020): 3341–3350. 32193608 10.1007/s00018-020-03489-9 PMC11105033

[25] J. Y. Lee , J. H. Park , H. J. Choi , et al., “LSD1 Demethylates HIF1α to Inhibit Hydroxylation and Ubiquitin‐Mediated Degradation in Tumor Angiogenesis,” Oncogene 36, no. 39 (2017): 5512–5521. 28534506 10.1038/onc.2017.158

[26] D. Kim , K. I. Kim , and S. H. Baek , “Roles of Lysine‐Specific Demethylase 1 (LSD1) in Homeostasis and Diseases,” Journal of Biomedical Science 28, no. 1 (2021): 41. 34082769 10.1186/s12929-021-00737-3 PMC8175190

[27] Y. Kim , H. J. Nam , J. Lee , et al., “Methylation‐Dependent Regulation of HIF‐1α Stability Restricts Retinal and Tumour Angiogenesis,” Nature Communications 7, no. 1 (2016): 10347. 10.1038/ncomms10347 PMC4735525 26757928

[28] S. J. Yang , Y. S. Park , J. H. Cho , et al., “Regulation of Hypoxia Responses by Flavin Adenine Dinucleotide‐Dependent Modulation of HIF‐1α Protein Stability,” EMBO Journal 36, no. 8 (2017): 1011–1028. 28279976 10.15252/embj.201694408 PMC5391145

[29] K. Doi , K. Murata , S. Ito , et al., “Role of Lysine‐Specific Demethylase 1 in Metabolically Integrating Osteoclast Differentiation and Inflammatory Bone Resorption Through Hypoxia‐Inducible Factor 1alpha and E2F1,” Arthritis and Rheumatology 74, no. 6 (2022): 948–960. 35077015 10.1002/art.42074 PMC9156537

[30] V. Janbandhu , V. Tallapragada , R. Patrick , et al., “Hif‐1a Suppresses ROS‐Induced Proliferation of Cardiac Fibroblasts Following Myocardial Infarction,” Cell Stem Cell 29, no. 2 (2022): 281–297.e12. 34762860 10.1016/j.stem.2021.10.009 PMC9021927

[31] K. Gabisonia , G. Prosdocimo , G. D. Aquaro , et al., “MicroRNA Therapy Stimulates Uncontrolled Cardiac Repair After Myocardial Infarction in Pigs,” Nature 569, no. 7756 (2019): 418–422. 31068698 10.1038/s41586-019-1191-6 PMC6768803

[32] J. Xiao , H. Liu , D. Cretoiu , et al., “miR‐31a‐5p Promotes Postnatal Cardiomyocyte Proliferation by Targeting RhoBTB1,” Experimental & Molecular Medicine 49, no. 10 (2017): e386. 29053138 10.1038/emm.2017.150 PMC5668467

[33] K. Thygesen , J. S. Alpert , A. S. Jaffe , et al., “Fourth Universal Definition of Myocardial Infarction (2018),” Circulation 138, no. 20 (2018): e618‐e651. 30571511 10.1161/CIR.0000000000000617

[34] Z. Wang , J. Zhu , Y. Liu , Z. Wang , X. Cao , and Y. Gu , “Tumor‐Polarized GPX3(+) AT2 Lung Epithelial Cells Promote Premetastatic Niche Formation,” Proceedings of the National Academy of Sciences of the United States of America 119, no. 32 (2022): e2201899119. 35914155 10.1073/pnas.2201899119 PMC9371733

[35] W. Manzanares , A. Biestro , M. H. Torre , F. Galusso , G. Facchin , and G. Hardy , “High‐Dose Selenium Reduces Ventilator‐Associated Pneumonia and Illness Severity in Critically Ill Patients With Systemic Inflammation,” Intensive Care Medicine 37, no. 7 (2011): 1120–1127. 21445641 10.1007/s00134-011-2212-6

[36] B. G. Kho , H.‐Y. Park , H.‐J. Cho , et al., “Glutathione Peroxidase 3 as a Biomarker of Recurrence After Lung Cancer Surgery,” Journal of Clinical Medicine 9, no. 12 (2020): 3801. 33255360 10.3390/jcm9123801 PMC7760369

[37] F. C. Simoes , T. J. Cahill , A. Kenyon , et al., “Macrophages Directly Contribute Collagen to Scar Formation During Zebrafish Heart Regeneration and Mouse Heart Repair,” Nature Communications 11, no. 1 (2020): 600. 10.1038/s41467-019-14263-2 PMC6992796 32001677

[38] C. J. A. Ramachandra , S. Hernandez‐Resendiz , G. E. Crespo‐Avilan , Y.‐H. Lin , and D. J. Hausenloy , “Mitochondria in Acute Myocardial Infarction and Cardioprotection,” eBioMedicine 57 (2020): 102884. 32653860 10.1016/j.ebiom.2020.102884 PMC7355051

[39] L. Li , M. Lu , Y. Peng , et al., “Oxidatively Stressed Extracellular Microenvironment Drives Fibroblast Activation and Kidney Fibrosis,” Redox Biology 67 (2023): 102868. 37690165 10.1016/j.redox.2023.102868 PMC10497796

[40] Y. Zeng , J. Huang , R. Guo , S. Cao , H. Yang , and W. Ouyang , “Identification and Validation of Metabolism‐Related Hub Genes in Idiopathic Pulmonary Fibrosis,” Frontiers in Genetics 14 (2023): 1058582. 36923791 10.3389/fgene.2023.1058582 PMC10010493

[41] G. Li , Y. Qin , Z. Cheng , et al., “Gpx3 and Egr1 Are Involved in Regulating the Differentiation Fate of Cardiac Fibroblasts Under Pressure Overload,” Oxidative Medicine and Cellular Longevity 2022, no. 2022 (2022): 1–21, 10.1155/2022/3235250. PMC9256463 35799890

[42] N. Guimarães‐Camboa , J. Stowe , I. Aneas , et al., “HIF1α Represses Cell Stress Pathways to Allow Proliferation of Hypoxic Fetal Cardiomyocytes,” Developmental Cell 33, no. 5 (2015): 507–521. 26028220 10.1016/j.devcel.2015.04.021 PMC4509618

[43] R. Bohuslavova , R. Cerychova , F. Papousek , et al., “HIF‐1α Is Required for Development of the Sympathetic Nervous System,” Proceedings of the National Academy of Sciences 116, no. 27 (2019): 13414–13423. 10.1073/pnas.1903510116 PMC6613092 31196952

[44] G. L. Semenza , “Angiogenesis Ischemic and Neoplastic Disorders,” Annual Review of Medicine 54, no. 1 (2003): 17–28. 10.1146/annurev.med.54.101601.152418 12359828

[45] G. Loor and P. T. Schumacker , “Role of Hypoxia‐Inducible Factor in Cell Survival During Myocardial Ischemia‐Reperfusion,” Cell Death and Differentiation 15, no. 4 (2008): 686–690. 18259200 10.1038/cdd.2008.13

[46] W. Dong , J. F. Weng , J. B. Zhu , et al., “CREB‐Binding Protein and HIF‐1α/β‐Catenin to Upregulate miR‐322 and Alleviate Myocardial Ischemia‐Reperfusion Injury,” FASEB Journal 37, no. 9 (2023): e22996. 37566526 10.1096/fj.202200596RRRRRR

[47] S. Ju , L. Lim , K. Wi , et al., “LRP5 Regulates HIF‐1α Stability via Interaction With PHD2 in Ischemic Myocardium,” International Journal of Molecular Sciences 22, no. 12 (2021): 6581. 34205318 10.3390/ijms22126581 PMC8235097

[48] L. L. Dunn , S. M. Y. Kong , S. Tumanov , et al., “Hmox1 (Heme Oxygenase‐1) Protects Against Ischemia‐Mediated Injury via Stabilization of HIF‐1α (Hypoxia‐Inducible Factor‐1α),” Arteriosclerosis, Thrombosis, and Vascular Biology 41, no. 1 (2020): 317–330, 10.1161/ATVBAHA.120.315393. 33207934

[49] A. T. Reddy , S. P. Lakshmi , A. Banno , and R. C. Reddy , “Role of GPx3 in PPARγ‐Induced Protection Against COPD‐Associated Oxidative Stress,” Free Radical Biology & Medicine 126 (2018): 350–357. 30118830 10.1016/j.freeradbiomed.2018.08.014 PMC6368849

