# Supplementary material for: GPX3 Overexpression Ameliorates Cardiac Injury Post Myocardial Infarction Through Activating LSD1/Hif1α Axis
Source: J Cell Mol Med. 2025 Feb 3;29(3):e70398. doi: 10.1111/jcmm.70398 (PMC11790353; doi:10.1111/jcmm.70398)
Supplement: Supplementary file 1 — Supplementary Tables 1‐2. [file JCMM-29-e70398-s001.docx]

**Supplemental Tables**

**Supplemental Table 1. The sequences of RT-PCR primers.**

| Primers | Sequences (5'–3') |
| --- | --- |
| GPX3-F-RAT | GGCTTTGTGCCTAATTTCCA |
| GPX3-R-RAT | CCCACCAGGAACTTCTCAAA |
| GPX3-F-MOUSE | CCTTTTAAGCAGTATGCAGGCA |
| GPX3-R-MOUSE | CAAGCCAAATGGCCCAAGTT |
| LSD1-F-RAT | CGCCACGGTCTTATCAACTT |
| LSD1-R-RAT | GCCAGAAACACCTGAGCCTA |
| Hif1a-F-RAT | CAACTGCCACCACTGATGAATC |
| Hif1a-R-RAT | ACCACTGTATGCTGATGCCTTAG |
| 18S-F-MOUSE | TAACGAACGAGACTCTGGCAT |
| 18S-R-MOUSE | CGGACATCTAAGGGCATCACAG |
| β-ACTIN-F-RAT | TAAAGACCTCTATGCCAACACAGT |
| β-ACTIN-R-RAT | CACGATGGAGGGGCCGGACTCATC |

**Supplemental Table 2. The differentially expressed genes (DEGs) in swine acute myocardial ischemia/reperfusion model**

| **protein** | **gene** | **gene_id** | **passed** | **imputed** | **sham1** | **sham2** | **sham3** | **IR1** | **IR2** | **IR3** |
| --- | --- | --- | --- | --- | --- | --- | --- | --- | --- | --- |
| F1SVB2 | RETSAT | 100519138 | TRUE | 0 | 436 | 437 | 345 | 155 | 161 | 161 |
| F1S2Q5 | ZC2HC1C | 100153971 | TRUE | 0 | 75 | 65 | 72 | 51 | 40 | 43 |
| A0A287ABC7 | PSMC3 | - | TRUE | 0 | 127 | 124 | 112 | 67 | 66 | 73 |
| F1SDF7 | LOC100156977 | 100156977 | TRUE | 0 | 593 | 540 | 440 | 205 | 217 | 236 |
| F1RK80 | TSSK1B | 102159240 | TRUE | 0 | 757 | 799 | 747 | 362 | 328 | 388 |
| I3LUW6 | FHIP1A | - | TRUE | 0 | 108 | 105 | 112 | 75 | 64 | 70 |
| A0A286ZT09 | CROT | - | TRUE | 0 | 126 | 122 | 125 | 66 | 74 | 81 |
| F1SPY4 | LOC100624149 | 100624149 | TRUE | 0 | 167 | 165 | 191 | 60 | 61 | 62 |
| K7GSK9 | PRKAG3 | - | TRUE | 0 | 330 | 320 | 290 | 163 | 151 | 206 |
| A0A1B2TT47 | DDX3Y | 100624590 | TRUE | 0 | 202 | 193 | 216 | 76 | 80 | 71 |
| A0A287AJ46 | EPHX2 | - | TRUE | 0 | 7,266 | 7,160 | 6,218 | 2,447 | 2,294 | 2,818 |
| F1SRQ0 | NDUFB2 | - | TRUE | 0 | 938 | 844 | 810 | 574 | 438 | 563 |
| A0A286ZJC9 | SORBS1 | - | TRUE | 0 | 147 | 134 | 129 | 73 | 55 | 73 |
| F1SHW5 | AGXT | - | TRUE | 0 | 145 | 202 | 170 | 86 | 99 | 105 |
| A0A5G2QA30 | SYNGR1 | 100626046 | TRUE | 0 | 163 | 168 | 149 | 104 | 87 | 109 |
| A0A287BA24 | GCSH | - | TRUE | 0 | 1,367 | 1,375 | 1,189 | 634 | 561 | 672 |
| O79876 | MT-CO1 | 808503 | TRUE | 0 | 1,072 | 1,182 | 959 | 585 | 459 | 598 |
| A0A287A792 | SLC7A6OS | - | TRUE | 0 | 200 | 162 | 234 | 69 | 69 | 74 |
| I3LLX4 | PLEKHB2 | - | TRUE | 0 | 395 | 400 | 343 | 218 | 207 | 251 |
| A0A287A972 | SLC25A29 | - | TRUE | 0 | 877 | 859 | 827 | 521 | 442 | 611 |
| F1S8S4 | ENAH | - | TRUE | 0 | 485 | 388 | 442 | 255 | 246 | 260 |
| D0VWV4 | SDHC | 100524676 | TRUE | 0 | 1,811 | 1,947 | 1,760 | 1,092 | 941 | 1,285 |
| A0A287B726 | IRS2 | - | TRUE | 0 | 198 | 189 | 190 | 125 | 114 | 140 |
| A0A287B105 | COX7B | - | TRUE | 0 | 1,020 | 1,154 | 1,026 | 539 | 452 | 604 |
| A5GZW8 | SDHD | 100048954 | TRUE | 0 | 3,566 | 3,910 | 3,274 | 2,012 | 1,798 | 2,453 |
| F1SUS8 | COA4 | 100514530 | TRUE | 0 | 550 | 469 | 488 | 133 | 126 | 177 |
| A0A286ZXL2 | PITPNM1 | - | TRUE | 0 | 81 | 74 | 76 | 47 | 48 | 56 |
| F1RFD4 | ATP5MK | - | TRUE | 0 | 911 | 848 | 811 | 509 | 441 | 555 |
| Q35915 | MT-ATP6 | 808506 | TRUE | 0 | 2,137 | 2,192 | 2,023 | 1,291 | 1,115 | 1,430 |
| A0A481BCG1 | MTARC2 | - | TRUE | 0 | 10,549 | 10,733 | 9,265 | 5,991 | 5,593 | 6,964 |
| I3LGW9 | USP13 | - | TRUE | 0 | 1,895 | 1,940 | 1,993 | 957 | 880 | 1,130 |
| A0A5G2R8S6 | IVD | - | TRUE | 0 | 12,286 | 12,253 | 12,237 | 8,250 | 7,279 | 8,780 |
| Q1W0Y2 | COX7C | 100037990 | TRUE | 0 | 7,147 | 6,151 | 7,580 | 4,041 | 3,364 | 4,243 |
| Q2HYU1 | CKMT2 | 733602 | TRUE | 0 | 55,430 | 47,351 | 48,731 | 31,596 | 28,684 | 37,062 |
| F1RLI6 | SLC25A46 | - | TRUE | 0 | 550 | 594 | 499 | 326 | 316 | 381 |
| A0A287AL59 | PCYT2 | - | TRUE | 0 | 1,362 | 1,144 | 1,298 | 809 | 890 | 807 |
| I3LAB7 | CYP27A1 | - | TRUE | 0 | 704 | 716 | 654 | 352 | 331 | 405 |
| F1SL15 | TRMT10C | 100522163 | TRUE | 0 | 1,200 | 1,115 | 1,169 | 686 | 620 | 754 |
| A0A5G2REF2 | ASPH | - | TRUE | 0 | 6,371 | 6,257 | 6,224 | 2,791 | 2,674 | 3,789 |
| F1RL20 | CHCHD10 | 100152325 | TRUE | 0 | 121 | 101 | 107 | 65 | 57 | 77 |
| A0A286ZU53 | SURF1 | - | TRUE | 0 | 2,599 | 2,513 | 2,484 | 1,598 | 1,421 | 1,799 |
| Q864V5 | FUNDC2 | 396564 | TRUE | 0 | 581 | 567 | 589 | 340 | 301 | 418 |
| A0A5G2QSG8 | MVK | - | TRUE | 0 | 954 | 1,157 | 940 | 602 | 551 | 709 |
| A0A5G2R6F6 | GATD3A | 100623793 | TRUE | 0 | 8,937 | 8,738 | 8,561 | 4,682 | 3,895 | 5,654 |
| A0A287ADJ1 | TECRL | - | TRUE | 0 | 2,653 | 2,593 | 2,752 | 1,560 | 1,501 | 1,896 |
| F1RGT4 | MCRIP2 | 100525039 | TRUE | 0 | 129 | 114 | 137 | 76 | 72 | 86 |
| Q8SPJ9 | COX7A1 | 399685 | TRUE | 0 | 5,322 | 5,040 | 5,594 | 3,154 | 2,668 | 3,670 |
| F6Q0K3 | NDUFB6 | - | TRUE | 0 | 7,619 | 7,665 | 7,283 | 4,808 | 4,257 | 5,457 |
| I3LGM4 | NDUFA11 | 100514033 | TRUE | 0 | 4,283 | 3,986 | 4,318 | 2,768 | 2,400 | 3,142 |
| I3L8Q5 | PECR | - | TRUE | 0 | 205 | 285 | 258 | 145 | 150 | 148 |
| F1S2X3 | ECHDC1 | - | TRUE | 0 | 7,444 | 7,636 | 7,149 | 4,704 | 4,075 | 5,321 |
| A0A5G2QKW6 | NDUFAF5 | 100520522 | TRUE | 0 | 3,745 | 3,799 | 3,596 | 2,200 | 1,865 | 2,630 |
| A0A286ZVG3 | LOC110257905 | - | TRUE | 0 | 230 | 232 | 243 | 122 | 114 | 119 |
| A0A287A9T9 | SDR39U1 | 100152844 | TRUE | 0 | 2,358 | 2,461 | 2,095 | 1,361 | 1,181 | 1,612 |
| F1SBU7 | LONP1 | - | TRUE | 0 | 12,052 | 11,499 | 12,452 | 7,156 | 6,667 | 8,395 |
| A0A287BFZ3 | WFS1 | - | TRUE | 0 | 2,164 | 2,055 | 2,102 | 1,228 | 1,247 | 1,608 |
| I3L5P4 | THNSL2 | - | TRUE | 0 | 1,316 | 1,276 | 1,234 | 823 | 769 | 900 |
| I3LER5 | COX4I1 | - | TRUE | 0 | 24,774 | 24,217 | 23,195 | 12,592 | 11,714 | 15,257 |
| F1SD77 | C2H19orf53 | 100526037 | TRUE | 0 | 570 | 562 | 494 | 288 | 271 | 359 |
| A0A287BAZ8 | AASS | - | TRUE | 0 | 4,911 | 5,455 | 4,597 | 2,325 | 2,412 | 2,896 |
| F1SLX5 | AASS | 100513962 | TRUE | 0 | 572 | 641 | 518 | 208 | 213 | 295 |
| A0A5G2R5P0 | APOO | - | TRUE | 0 | 4,311 | 4,286 | 4,272 | 2,735 | 2,428 | 3,073 |
| A0A286ZX57 | PPM1K | - | TRUE | 0 | 571 | 554 | 594 | 376 | 321 | 428 |
| F1RPU8 | MPC2 | - | TRUE | 0 | 3,826 | 3,816 | 3,885 | 2,362 | 2,182 | 2,673 |
| P00258 | FDX1 | 397133 | TRUE | 0 | 1,566 | 1,624 | 1,602 | 977 | 982 | 1,108 |
| A0A286ZWQ9 | LOC100739365 | 100739365 | TRUE | 0 | 11,825 | 10,813 | 11,764 | 6,660 | 5,988 | 7,453 |
| A0A5G2QBF4 | CMC1 | 102164646 | TRUE | 0 | 1,319 | 1,276 | 1,358 | 859 | 753 | 983 |
| A0A286ZQZ7 | CHGB | - | TRUE | 0 | 1,218 | 1,518 | 1,258 | 636 | 633 | 698 |
| A0A287B7Q9 | ACSS1 | - | TRUE | 0 | 2,237 | 1,919 | 2,569 | 1,092 | 1,277 | 1,392 |
| I3LUR5 | ACAD9 | - | TRUE | 0 | 11,468 | 11,085 | 11,835 | 6,788 | 6,651 | 7,000 |
| F1S451 | LDHD | - | TRUE | 0 | 2,354 | 2,559 | 2,556 | 1,010 | 920 | 1,251 |
| I3L5X7 | RBM3 | 100627807 | TRUE | 0 | 316 | 290 | 324 | 210 | 168 | 212 |
| K7GP41 | MPP1 | 100517992 | TRUE | 0 | 1,364 | 1,147 | 1,356 | 772 | 854 | 766 |
| A0A5G2RHS1 | APOOL | - | TRUE | 0 | 5,575 | 5,200 | 4,861 | 3,554 | 3,229 | 3,560 |
| I3LTJ4 | SDHAF3 | - | TRUE | 0 | 622 | 583 | 657 | 399 | 392 | 449 |
| A0A286ZKD8 | CPLX1 | - | TRUE | 0 | 485 | 470 | 434 | 271 | 244 | 330 |
| F1S8L9 | HNRNPU | - | TRUE | 0 | 273 | 239 | 243 | 144 | 142 | 124 |
| A0A287AQB8 | MGST2 | - | TRUE | 0 | 360 | 357 | 357 | 223 | 227 | 247 |
| A0A480K1R7 | LOC100517502 | - | TRUE | 0 | 253 | 211 | 220 | 125 | 130 | 136 |
| A0A5G2QPK8 | CPLX1 | - | TRUE | 0 | 212 | 216 | 182 | 86 | 76 | 115 |
| A0A5G2RFX9 | ACAD11 | - | TRUE | 0 | 438 | 455 | 422 | 277 | 258 | 276 |
| A0A5G2QMX0 | H2AX | - | TRUE | 0 | 346 | 317 | 402 | 574 | 643 | 587 |
| A0A287BI36 | PDLIM5 | - | TRUE | 0 | 2,534 | 2,251 | 2,545 | 1,586 | 1,445 | 1,655 |
| A0A287B7U0 | RPLP2 | 100513643 | TRUE | 0 | 1,379 | 1,362 | 1,415 | 2,184 | 2,263 | 1,903 |
| F1RKM0 | LMNB1 | 100513342 | TRUE | 0 | 11,395 | 10,855 | 10,665 | 17,776 | 18,561 | 14,309 |
| I3LJP2 | SELENOP | - | TRUE | 0 | 762 | 713 | 691 | 1,242 | 1,346 | 998 |
| A0A288CFV0 | L3MBTL1 | - | TRUE | 0 | 1,359 | 1,327 | 1,337 | 2,199 | 2,308 | 1,814 |
| A0A287AX59 | RPL37 | - | TRUE | 0 | 3,245 | 3,200 | 3,290 | 5,174 | 5,176 | 4,673 |
| A0A5G2QTH6 | MAOA | - | TRUE | 0 | 843 | 880 | 744 | 1,296 | 1,349 | 1,238 |
| A0A5G2QS80 | CCN1 | 100153791 | TRUE | 0 | 88 | 79 | 95 | 139 | 141 | 153 |
| A0A287BM33 | ERLEC1 | - | TRUE | 0 | 185 | 207 | 196 | 282 | 338 | 284 |
| A0A287B5Z8 | NOP10 | 100156164 | TRUE | 0 | 272 | 270 | 285 | 448 | 433 | 371 |
| A0A287AIW0 | CD55 | - | TRUE | 0 | 82 | 84 | 81 | 160 | 151 | 117 |
| A0A287B771 | RPS4X | 397611 | TRUE | 0 | 6,344 | 5,881 | 6,514 | 9,938 | 9,642 | 8,672 |
| I3LC14 | ADAMTSL2 | 100628047 | TRUE | 0 | 132 | 135 | 125 | 209 | 239 | 190 |
| A0A075B7J0 | - | - | TRUE | 0 | 907 | 828 | 815 | 1,413 | 1,537 | 1,228 |
| K7GRN9 | NIBAN1 | 100516994 | TRUE | 0 | 5,363 | 5,261 | 5,134 | 8,340 | 9,036 | 7,320 |
| Q9GMB0 | RPN1 | 397606 | TRUE | 0 | 5,124 | 5,253 | 5,245 | 8,210 | 8,946 | 7,145 |
| P62901 | RPL31 | 100737826 | TRUE | 0 | 1,696 | 1,708 | 1,873 | 2,619 | 2,932 | 2,461 |
| A0A287AIJ3 | GPX3 | - | TRUE | 0 | 1,999 | 2,201 | 1,936 | 2,978 | 3,723 | 3,388 |
| A0A287AP95 | - | - | TRUE | 0 | 546 | 528 | 534 | 1,204 | 1,386 | 909 |
| P06867 | PLG | 733660 | TRUE | 0 | 1,111 | 1,094 | 1,004 | 1,765 | 2,085 | 1,508 |
| F1RJJ0 | SRSF9 | - | TRUE | 0 | 392 | 408 | 370 | 568 | 686 | 538 |
| F1S3J0 | ACOT6 | 100152868 | TRUE | 0 | 420 | 367 | 412 | 215 | 173 | 239 |
| F1SDX6 | TGM2 | - | TRUE | 0 | 12,159 | 12,101 | 12,113 | 19,410 | 20,256 | 18,857 |
| K7GP63 | LOC100623540 | 100515520 | TRUE | 0 | 307 | 292 | 318 | 605 | 530 | 429 |
| A0A287AA45 | HNMT | 100624677 | TRUE | 0 | 728 | 742 | 644 | 1,166 | 1,159 | 1,108 |
| A0A287B929 | RPL13A | 780432 | TRUE | 0 | 2,830 | 3,244 | 2,464 | 4,796 | 4,647 | 5,062 |
| F1SLY7 | CNPY2 | 100151912 | TRUE | 0 | 1,290 | 1,269 | 1,216 | 2,016 | 2,344 | 1,709 |
| F1RMM0 | CAVIN3 | 100513556 | TRUE | 0 | 2,197 | 2,163 | 2,055 | 3,937 | 3,137 | 3,022 |
| A0A5G2R1D5 | RPL10 | - | TRUE | 0 | 2,866 | 2,819 | 3,014 | 4,660 | 4,522 | 4,130 |
| P04366 | AMBP | - | TRUE | 0 | 2,723 | 2,513 | 2,566 | 4,230 | 4,613 | 3,469 |
| F1RYT3 | SCARB2 | 414407 | TRUE | 0 | 2,075 | 2,244 | 2,064 | 3,674 | 3,894 | 2,956 |
| A0A5G2QJH6 | RBM28 | - | TRUE | 0 | 145 | 158 | 141 | 232 | 235 | 208 |
| A0A287AW71 | - | - | TRUE | 0 | 1,507 | 1,414 | 1,619 | 2,704 | 2,667 | 2,287 |
| A0A287ALJ6 | - | - | TRUE | 0 | 2,612 | 2,394 | 2,491 | 5,387 | 6,562 | 4,123 |
| A0A287BAB3 | - | - | TRUE | 0 | 3,413 | 3,139 | 3,126 | 5,443 | 6,409 | 4,534 |
| P80310 | S100A12 | 100301483 | TRUE | 0 | 428 | 394 | 472 | 1,103 | 958 | 706 |
| F1RN41 | F10 | - | TRUE | 0 | 1,215 | 1,172 | 1,279 | 1,879 | 2,114 | 1,637 |
| A0A5G2QUE0 | SERPINC1 | - | TRUE | 0 | 6,397 | 5,836 | 5,721 | 10,002 | 10,498 | 8,032 |
| F1SFI6 | FETUB | 100517609 | TRUE | 0 | 2,053 | 1,975 | 1,979 | 3,888 | 4,422 | 3,173 |
| A0A5G2QGH3 | SUMO2 | - | TRUE | 0 | 220 | 226 | 222 | 340 | 368 | 299 |
| A0A287AFU9 | LOC110256441 | - | TRUE | 0 | 259 | 259 | 268 | 580 | 482 | 482 |
| A0A287AYW8 | SERPINE2 | - | TRUE | 0 | 956 | 880 | 1,073 | 2,317 | 2,326 | 1,756 |
| A0A286ZLN6 | - | - | TRUE | 0 | 405 | 386 | 413 | 1,015 | 1,126 | 791 |
| Q9TUQ3 | C7 | 397526 | TRUE | 0 | 1,233 | 1,282 | 1,264 | 2,303 | 2,684 | 1,864 |
| P09571 | TF | - | TRUE | 0 | 34,175 | 30,715 | 31,460 | 55,225 | 64,802 | 47,327 |
| F1SH96 | ITIH1 | - | TRUE | 0 | 842 | 743 | 742 | 1,711 | 1,890 | 1,285 |
| A0A5G2R1M5 | PTGIS | - | TRUE | 0 | 537 | 663 | 563 | 1,036 | 1,068 | 830 |
| F1SMW8 | SERPINB10 | 100154994 | TRUE | 0 | 171 | 174 | 172 | 289 | 330 | 238 |
| A0A5G2R416 | TGM2 | - | TRUE | 0 | 338 | 344 | 366 | 632 | 752 | 615 |
| A0A5G2Q8T9 | RPL3 | - | TRUE | 0 | 3,001 | 3,170 | 3,016 | 6,501 | 7,714 | 4,777 |
| F1RF14 | CES1 | - | TRUE | 0 | 838 | 782 | 764 | 1,358 | 1,098 | 1,197 |
| A0A287BGN0 | COX6A1 | 100152232 | TRUE | 0 | 178 | 181 | 178 | 269 | 268 | 281 |
| F1S815 | ROR1 | - | TRUE | 0 | 149 | 187 | 154 | 329 | 350 | 254 |
| F6Q697 | S100A9 | - | TRUE | 0 | 439 | 441 | 500 | 902 | 767 | 714 |
| F6Q6H5 | MDK | - | TRUE | 0 | 462 | 426 | 493 | 920 | 1,137 | 744 |
| P79263 | ITIH4 | 396799 | TRUE | 0 | 840 | 824 | 900 | 1,357 | 1,413 | 1,208 |
| A0A287AD38 | TPM2 | - | TRUE | 0 | 1,471 | 1,341 | 1,534 | 5,529 | 4,168 | 2,822 |
| A0A287B310 | STOM | - | TRUE | 0 | 2,022 | 2,050 | 2,004 | 3,234 | 3,190 | 3,214 |
| F1RG45 | AGT | - | TRUE | 0 | 1,269 | 1,244 | 1,240 | 2,422 | 2,551 | 1,966 |
| P32195 | NPG2 | 100170125 | TRUE | 0 | 705 | 681 | 776 | 2,020 | 1,839 | 1,382 |
| A0A286ZVB7 | DPT | 100516366 | TRUE | 0 | 2,024 | 2,117 | 2,003 | 6,111 | 5,213 | 3,704 |
| A0A287AQ20 | CFI | 100516921 | TRUE | 0 | 2,921 | 2,801 | 2,859 | 5,621 | 6,826 | 4,648 |
| A0A5G2QEW1 | ITIH4 | - | TRUE | 0 | 10,693 | 10,547 | 10,545 | 22,230 | 24,777 | 17,323 |
| F1SFI7 | AHSG | 397585 | TRUE | 0 | 3,884 | 3,733 | 3,631 | 7,747 | 9,360 | 5,905 |
| F2Z546 | RPL19 | - | TRUE | 0 | 2,327 | 2,572 | 2,597 | 5,076 | 5,558 | 4,715 |
| A0A287BH90 | C9 | - | TRUE | 0 | 3,275 | 3,048 | 2,969 | 5,516 | 6,196 | 4,390 |
| P02543 | VIM | 100522394 | TRUE | 0 | 22,067 | 21,696 | 20,654 | 49,914 | 57,316 | 43,157 |
| A0A5G2QN94 | PRPS2 | - | TRUE | 0 | 126 | 140 | 125 | 200 | 230 | 175 |
| A0A286ZMH6 | LOC110259328 | - | TRUE | 0 | 174 | 176 | 175 | 232 | 275 | 288 |
| A0A5G2R4M3 | ITIH3 | - | TRUE | 0 | 3,399 | 3,587 | 3,090 | 8,439 | 8,514 | 6,856 |
| P51779 | CFD | 396877 | TRUE | 0 | 102 | 100 | 112 | 171 | 184 | 142 |
| F1SE20 | PLAT | - | TRUE | 0 | 431 | 463 | 441 | 831 | 861 | 733 |
| A0A5G2QN76 | CD44 | - | TRUE | 0 | 149 | 167 | 170 | 357 | 418 | 270 |
| A0A287BPP0 | - | - | TRUE | 0 | 352 | 353 | 356 | 919 | 1,130 | 726 |
| A0A5G2QFI9 | SAA2 | - | TRUE | 0 | 411 | 433 | 439 | 698 | 777 | 632 |
| A0A5G2RAY2 | POFUT2 | - | TRUE | 0 | 559 | 537 | 520 | 1,102 | 1,265 | 816 |
| A0A287A1M4 | - | - | TRUE | 0 | 3,487 | 3,303 | 3,411 | 5,664 | 6,713 | 5,231 |
| A0A0H5ANC0 | OGN | 106509723 | TRUE | 0 | 3,120 | 3,583 | 3,117 | 7,439 | 9,439 | 7,762 |
| I3LFP3 | VCAN | - | TRUE | 0 | 3,371 | 4,079 | 3,406 | 9,813 | 12,670 | 7,330 |
| Q9XSD9 | DCN | 396957 | TRUE | 0 | 7,052 | 7,716 | 7,240 | 20,994 | 27,812 | 15,356 |
| Q8WMQ3 | CD9 | 397067 | TRUE | 0 | 221 | 242 | 220 | 627 | 725 | 404 |
| F1SUZ4 | STIM1 | - | TRUE | 0 | 167 | 207 | 181 | 295 | 340 | 268 |
| F1S981 | SPON1 | - | TRUE | 0 | 2,322 | 2,344 | 2,615 | 5,599 | 7,305 | 4,617 |
| Q8SPS7 | HP | 397061 | TRUE | 0 | 6,794 | 6,322 | 6,329 | 11,697 | 14,676 | 9,764 |
| F1SJM0 | PTX3 | - | TRUE | 0 | 110 | 118 | 120 | 291 | 243 | 213 |
| A0A286ZXQ0 | ATL3 | - | TRUE | 0 | 499 | 551 | 516 | 1,330 | 1,542 | 904 |
| A0A5G2R0Y2 | COL6A6 | - | TRUE | 0 | 4,626 | 5,055 | 4,301 | 14,777 | 14,110 | 9,822 |
| F1SUE4 | ASPN | 100511749 | TRUE | 0 | 5,313 | 4,736 | 5,741 | 14,421 | 21,508 | 11,833 |
| F1SQ09 | LUM | 100152607 | TRUE | 0 | 4,436 | 4,900 | 4,767 | 12,837 | 16,250 | 9,240 |
| I3LS97 | PUDP | - | TRUE | 0 | 517 | 452 | 532 | 1,106 | 1,004 | 1,040 |
| F1S3G7 | COL5A3 | - | TRUE | 0 | 127 | 138 | 122 | 353 | 314 | 227 |
| A0A286ZSL2 | AIF1L | - | TRUE | 0 | 278 | 279 | 360 | 1,201 | 905 | 649 |
| I3LF61 | CYP4F8 | - | TRUE | 0 | 211 | 214 | 209 | 368 | 401 | 413 |
| A0A287ATT2 | - | - | TRUE | 0 | 2,088 | 1,946 | 1,993 | 5,115 | 6,576 | 4,189 |
| A0A287BB68 | MYH11 | - | TRUE | 0 | 10,591 | 9,826 | 13,281 | 66,136 | 43,777 | 29,074 |
| A0A287B9B9 | IL1RAP | 100628318 | TRUE | 0 | 105 | 108 | 103 | 157 | 194 | 149 |
| F1SN68 | ORM1 | 396901 | TRUE | 0 | 2,318 | 2,168 | 2,227 | 5,463 | 7,248 | 4,176 |
| F1S021 | COL5A1 | - | TRUE | 0 | 410 | 474 | 385 | 1,651 | 2,237 | 1,006 |
| F1RPC8 | CRYM | 100525333 | TRUE | 0 | 244 | 282 | 238 | 872 | 807 | 568 |
| F1S9C0 | SAA2 | - | TRUE | 0 | 109 | 104 | 104 | 306 | 389 | 229 |
| I3L7W9 | - | - | TRUE | 0 | 76 | 83 | 72 | 285 | 393 | 176 |
| A0A287BGF5 | LOC110255206 | - | TRUE | 0 | 1,228 | 1,373 | 1,710 | 4,411 | 5,857 | 3,213 |
| I3LQS2 | NT5C | 100526240 | TRUE | 0 | 113 | 120 | 128 | 189 | 229 | 184 |
| Q29558 | ME1 | - | TRUE | 0 | 56 | 58 | 57 | 100 | 106 | 93 |
| A0A5G2QVS9 | MYL1 | - | TRUE | 0 | 694 | 858 | 932 | 4,145 | 3,007 | 2,944 |
| A0A287A481 | PLG | - | TRUE | 0 | 9,519 | 9,679 | 8,804 | 14,626 | 18,680 | 13,717 |
| K7GPT9 | CFB | - | TRUE | 0 | 7,513 | 7,402 | 7,518 | 12,764 | 13,826 | 10,277 |
| I3LQ79 | MVP | - | TRUE | 0 | 6,407 | 6,823 | 7,119 | 13,270 | 15,101 | 10,501 |
| F1SRF0 | HHATL | - | TRUE | 0 | 3,395 | 3,497 | 3,200 | 1,976 | 1,743 | 2,298 |
